# Semaphorin 7a is protective through immune modulation during acetaminophen-induced liver injury

**DOI:** 10.1186/s12950-025-00429-x

**Published:** 2025-03-20

**Authors:** Eilidh J. Livingstone, Jennifer A. Cartwright, Lara Campana, Philip J. Starkey Lewis, Benjamin J. Dwyer, Rhona Aird, Tak Yung Man, Matthieu Vermeren, Adriano Giorgio Rossi, Luke Boulter, Stuart John Forbes

**Affiliations:** 1 Centre for Regenerative Medicine, Institute for Regeneration and Repair, University of Edinburgh, Edinburgh, UK; 2 Centre for Inflammation Research, Institute for Regeneration and Repair, University of Edinburgh, Edinburgh, UK; 3 The Royal (Dick) School of Veterinary Studiesand Theaq , Roslin Institute, University of Edinburgh, Edinburgh, UK; 4 MRC Human Genetics Unit, Institute of Genetics and Molecular Medicine, University of Edinburgh, Edinburgh, EH4 2XU UK

**Keywords:** Sema7a, Paracetamol, APAP, APAP-induced liver injury, Macrophages, Neutrophils

## Abstract

**Background and Aim:**

Acetaminophen (APAP) induced acute liver injury (ALI), the leading cause acute liver failure in the western world, has limited treatment options. APAP toxicity results in massive hepatic necrosis and secondary infiltrating monocytes and neutrophils, which contribute to pathogenesis. Semaphorin 7a (Sema7a), a chemoattractant and modulator of monocytes and neutrophils, is a potential therapeutic target in other conditions, but its role in APAP-ALI is unexplored.

**Methods:**

Wild-type (WT) and Sema7a knockout (KO) mice were examined during APAP-ALI. Serum liver function tests, histological analysis and cellular localisation of Sema7a and its receptors, Plexin C1 and Integrin β1, were examined. Serum cytokines were quantified, tissue macrophages and neutrophils were localised, and in vivo phenotype, including phagocytosis, was assessed by immunohistochemistry and flow cytometry.

**Results:**

Sema7a was expressed by HNF4α + peri-necrotic hepatocytes circumferentially during APAP-ALI injury phases, and serum concentrations were increased, and correlated with hepatic injury. Sema7a KO mice had increased circulating inflammatory cytokines and significantly less hepatic F4/80 + macrophages, a cell type required for hepatic repair. Sema7a KO mice had higher necrotic area neutrophils, and increased neutrophil chemoattractant CXCL1. Without Sema7a expression, mice displayed increased necrosis and liver injury markers compared to Sema7a WT mice. Without peri-necrotic hepatocyte Sema7a expression, we also identified increased cell death and hepatic cellular stress outside of necrosis.

**Conclusion:**

We have identified a novel protective role of Sema7a during injury phases of APAP-ALI. Without peri-necrotic hepatocyte Sema7a expression and secretion, there is increased inflammation, time specific worsened hepatic necrosis and increased hepatic cell stress and death outside of the necrotic zone.

**Graphical Abstract:**

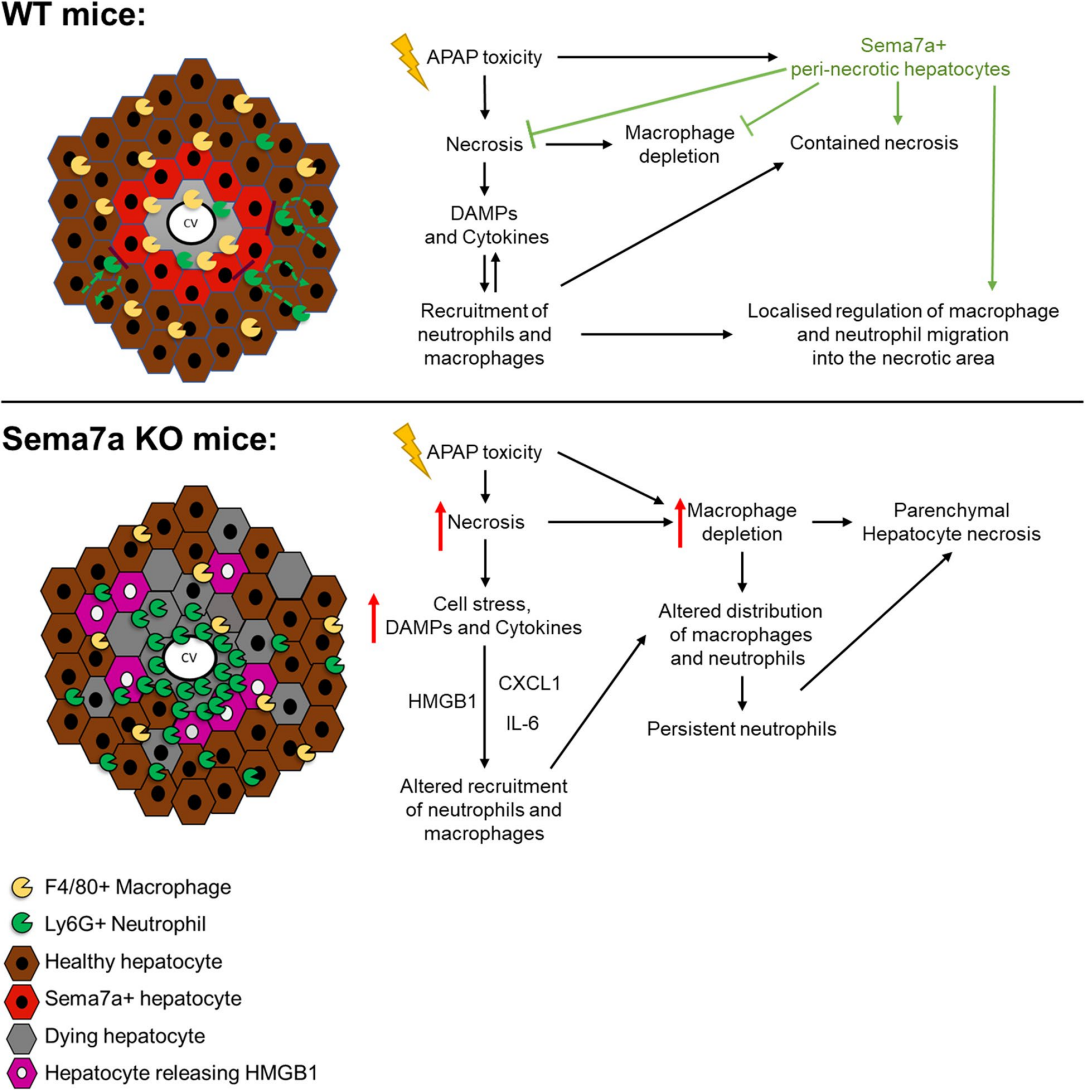

**Supplementary Information:**

The online version contains supplementary material available at 10.1186/s12950-025-00429-x.

## Synopsis

Semaphorin 7a (Sema7a) is expressed on hepatocytes in a circular ring surrounding necrotic cells during acute liver injury, and circulatory levels correlate with damage. Without peri-necrotic Sema7a and secretion, there is increased hepatic inflammation, and worsened necrosis, cell stress and death.

## Background

Acetaminophen (APAP) is a widely used analgesic and antipyretic medication, which is safe at therapeutic doses but overdose causes 46% of acute liver failure (ALF) in Western countries, and it remains the commonest cause of ALF-induced death [1–3]. APAP-induced acute liver injury (APAP-ALI) causes systemic inflammation, which can lead to multiorgan dysfunction and sepsis [4, 5]. The current pharmacological therapy for APAP-ALI, N-acetylcysteine (NAC), is effective if given within 10 h of APAP overdose ingestion [6]. Otherwise, only supportive care is available. Liver transplantation is considered for patients that develop liver failure, but suitable donor organs are not always available [7, 8]. Therefore, alternative therapeutic strategies are required to limit hepatic injury and promote recovery. One potential therapeutic avenue is to manipulate the innate immune system to limit damage and promote tissue recovery.

Semaphorins are a diverse family of highly conserved signalling proteins, that perform a variety of functions from neurogenesis axon guidance to bone homeostasis and immune responses [9–12]. Semaphorin 7a (Sema7a) is an immune semaphorin that modulates immune processes such as inflammatory infiltration, immune cell interactions, activation and suppression [10, 13, 14]. Sema7a is the only semaphorin connected to the membrane via a glycosylphosphatidylinositol linker, and it is expressed by neurones, immune cells and activated hepatic stellate cells [9, 15, 16]. Sema7a has two receptors Integrin β1 and Plexin C1 [17, 18], and promotes immune cell migration, such as facilitating neutrophil pulmonary transmigration [18, 19], or acting as a chemoattractant for monocytes and macrophages [20, 21]. Sema7a has cumulative evidence for involvement in autoimmune and inflammatory diseases [15, 20, 22] and anti-Sema7a treatment has shown efficacy for some of these conditions [23, 24]. More recent reports indicate a role in reducing inflammation in acute tissue damage [25, 26] but its actions have not been evaluated in APAP-ALI.

Despite conflicting literature, there is now substantial evidence that macrophages play a key role in resolving injury and inflammation following ALI [27–30], with poor outcomes from APAP-ALI correlating with blood monocytopenia [31]. Macrophages are highly plastic cells which acquire distinct phenotypes depending on molecular cues in their microenvironment [32, 33]. Ly6C^hi^ macrophages show a pro-inflammatory phenotype, whereas Ly6C^lo^ macrophages are considered restorative and promote restoration of the liver after CCl_4_ injury [34]. Sema7a can influence macrophage behaviour in a receptor-dependent manner and both pro-reparative and proinflammatory phenotypes have been documented [15, 22, 26].

APAP-ALI causes hepatocyte necrosis and the release of danger-associated-molecular patterns (DAMPs). DAMPs activate Kupffer cells (KCs), the liver resident macrophage, to release cytokines, which activate the innate immune system. This causes monocytes, macrophages and neutrophils to infiltrate the liver [33]. During APAP-ALI, KCs are depleted and monocyte-derived macrophages infiltrate the liver in large numbers [32]. Restorative Ly6C^lo^ macrophages have been shown to be crucial for appropriate liver repair via phagocytosis [35, 36] and secretion of IL-10 to reduce inflammation [28]. Macrophage ablation delays repair [29, 32, 37] and stimulating macrophage proliferation, or delivering alternatively-activated macrophages can promote liver regeneration [38, 39]. Neutrophils have controversial roles in APAP-ALI, with reports indicating they contribute to injury, or tissue repair [40–44]. Since Sema7a influences macrophage phenotype and has involvements in neutrophil migration, we tested whether Sema7a has a hepatoprotective role during acute liver injury and repair.

Using a genetic ablation approach, we describe the local and temporal effects of Sema7a during APAP-ALI, detailing Sema7a expression on peri-necrotic hepatocytes during injury and repair, and its importance to limit hepatocyte damage and reduce inflammation. We report that Sema7a KO mice have reduced hepatic F4/80 + macrophages, higher numbers of neutrophils within necrotic areas, and increased pro-inflammatory serum cytokine concentrations, alongside increased spread of tissue damage. In conclusion, we report that Sema7a has a protective role in APAP-ALI and modulates innate immune cells to limit the damage caused by APAP-ALI.

## Methods

All authors had access to the study data and had reviewed and approved the final manuscript.

### In vivo experiments

#### Animals

C57BL6/J or Sema7a^−/−^ mice (Jackson Laboratory, C57BL6/J background) were housed in specific pathogen-free environment and kept under standard conditions with a 12 h day/night cycle and access to food and water ad libitum. Mouse genotyping was performed by a commercial laboratory (Transnetyx). All animal experiments were carried out under procedural guidelines, severity protocols and with ethical permission from the University of Edinburgh Animal Welfare and Ethical Review Body and the Home Office (UK), licence numbers 70/7847 and P231C5F81.

#### APAP experiments

9–12 week old male mice were fasted for 12 h then intra-peritoneally (i.p.) injected with 350 mg/kg APAP (Sigma)), dissolved in sterile saline, n ≥ 6 / group. Controls were injected with an equivalent volume of sterile saline. Mice were kept in a 28 °C heat box, and closely monitored for the duration of the experiment.

Mice were humanely euthanised and blood was collected by cardiac puncture, into EDTA tubes for flow cytometry analysis, or clotted overnight at 4 °C then centrifuged for 10 min, 8,000 × g at 4 °C and serum collected. Livers were perfused with PBS and snap frozen or fixed in formalin overnight and paraffin embedded (FFPE).

Non-haemolysed serum liver function tests (LFTs) were performed by Dr Forbes Howie at the QMRI, University of Edinburgh according to manufacturer instructions (Alpha Laboratories). Kit instruction were adapted for use in the Cobas Fara or Cobas Mira analyser (Roche).

### Immunohistochemistry

Frozen sections were fixed for 20 min with ice cold Methanol: Acetone. 4 µm-thick FFPE sections were dewaxed and rehydrated before 15 min heat mediated antigen retrieval then permeabilised in PBS 0.1% Tween 20 (PBST). For IF sections were blocked for 30 min at RT with Protein Block (Spring Bio), then incubated overnight with the primary antibody at 4 °C, then incubated with secondary antibodies for 1 h RT. Sections were stained with DAPI (1:1,000) and mounted with fluoromount (Southern Biotech).

**Fig. 1:**
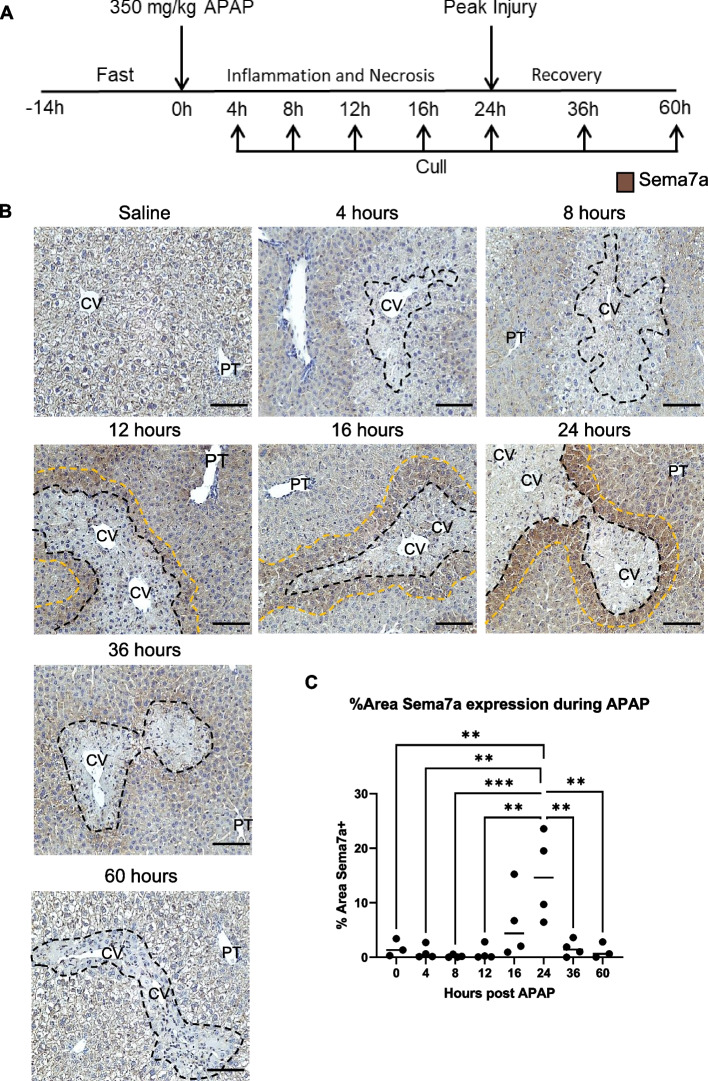
Sema7a is expressed on peri-necrotic cells during APAP-ALI. **A **Experimental schematic; WT mice received 350 mg/kg APAP i.p. and tissues collected during injury (< 24 h) and recovery/repair (> 24 h). **B** Sema7a expression during the APAP time course study. Black dashes outline necrotic areas. Yellow dashes outline Sema7a positive peri-necrotic cells. (C) Percentage area of Sema7a positivity, calculated from Image J, averaged from 6 fields of view/ mouse, 24 h expression highest *p* = 0.0005 ANOVA, Tukey's multiple comparisons test 0 vs 24 h *p* = 0.0057. Each datapoint represents an individual mouse. Scale bars 100 µm. CV, central vein; PT, portal tract; S7a, Sema7a. n ≥ 3 mice/time point

**Fig. 2:**
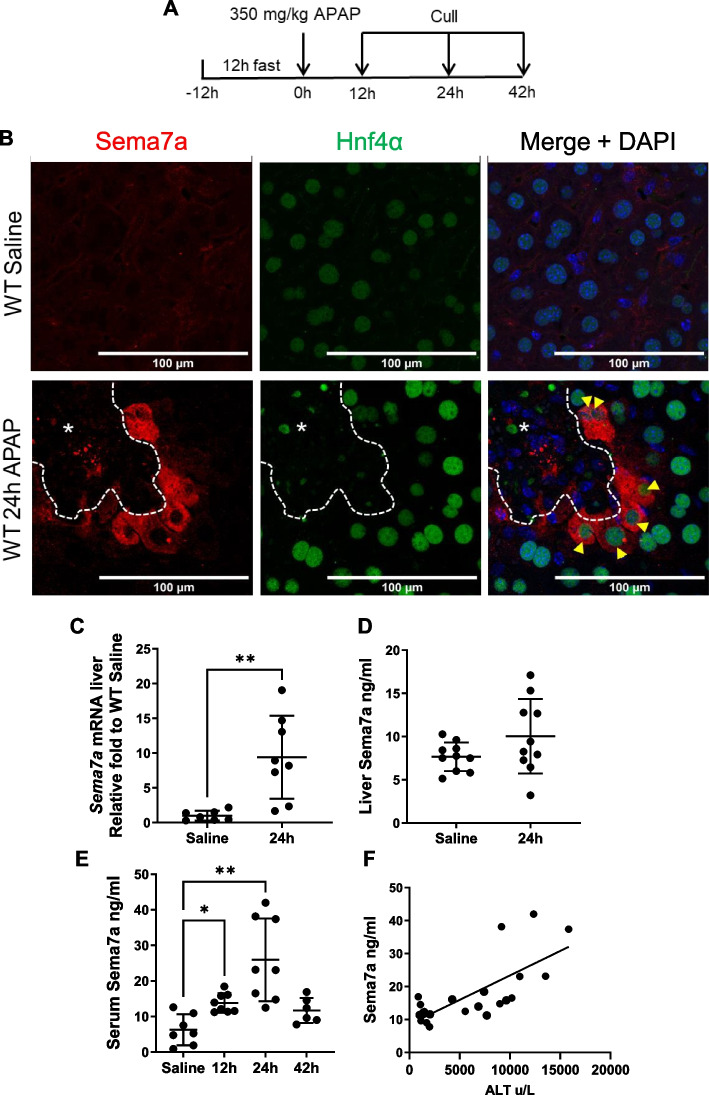
Sema7a is expressed by hepatocytes and correlates with serum ALT activity (**A**) Experimental schematic; WT mice received 350 mg/kg APAP i.p and tissues collected during injury (12 and 24 h) and repair (42 h) (**B**) Representative immunofluorescent labelled hepatic sections, Sema7a (red) and Hnf4α (green) from healthy mice (top) and at 24 h APAP-ALI (bottom). Yellow arrows, Sema7a + Hnf4a + hepatocytes. White dashed line and * indicate necrotic areas. **C** Whole liver lysate expression of Sema7a qRT-PCR, t-test, Welch’s correction (*p* = 0.0053); and (**D**) ELISA, unpaired t-test (*p* = 0.13). **E** Sema7a ELISA on serum during an APAP-ALI time course, Brown-Forsythe ANOVA (*P* = 0.0007), Dunnett’s comparisons Saline vs 12 h (*p* = 0.0149), Saline vs 24 h (*p* = 0.0089). **F** Correlation of Sema7a levels and ALT in serum. Linear Regression R2 = 0.53, *P* = 0.0001. Scale bars 100 µm. Each datapoint represents an individual mouse. * *p* < 0.05; ** *p* < 0.01. n ≥ 6 mice/ time point

For 3,3'-diaminobenzidine (DAB) stains, FFPE sections were sequentially blocked at RT with Bloxall (Vector) for 15 min, then Avidin and Biotin (Invitrogen) for 10 min each, followed by 30 min Protein Block. Sections were stained overnight at 4 °C with the primary antibody, followed by the secondary antibody for 1 h at RT. Slides were blocked with R.T.U. VECTASTAIN Elite ABC reagent (Vector) for 30 min, before detection with DAB (DAKO). Slides were counterstained with haematoxylin before dehydration and mounting. All primary antibodies, their required antigen retrieval and dilution is provided in Supplementary Table 1 & 2. Isotype controls can be seen in Supplementary Fig. 1, Supplementary Materials & Methods Figs. 1 and 2.

**Fig. 3:**
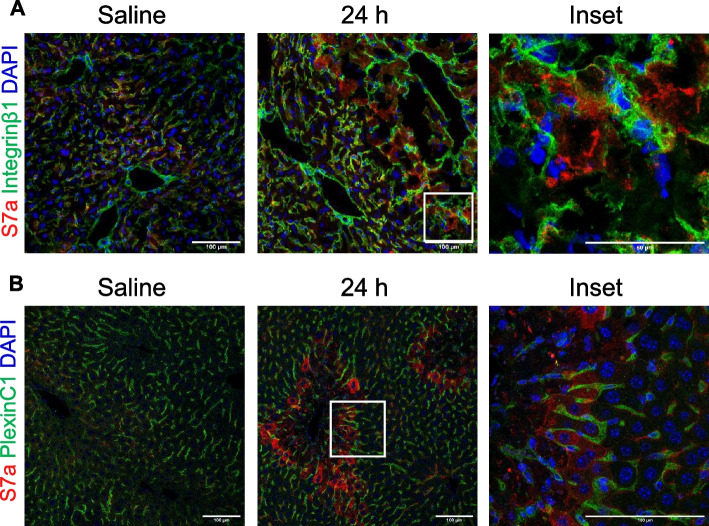
Expression of Sema7a and its Receptors PlexinC1 and Integrin β1 Results from 24 h post 350 mg/kg APAP or saline treated WT mice, Scale bars 100 µm. DAPI counterstain, n ≥ 8 mice/group, (**A**)Representative immunofluorescent labelled hepatic sections Sema7a (red) and Integrin β1 (green) with insert showing proximity of cell to cell expression, but no colocalization. **B** Representative immunofluorescent labelled hepatic sections Sema7a (red) and Plexin C1 (green) with insert showing proximity of cell to cell expression, but no colocalization

Haematoxylin and Eosin (H&E) staining and block processing was performed by SuRF Histology at the QMRI, University of Edinburgh. Slides were scanned with the Vectra Polaris multispectral slide scanner, and necrotic areas were segmented and quantified using inForm 2.4 (Perkin Elmer) software after tissue specific training, using eight 10 × FOV per mouse liver, at 1 μm resolution, as shown in Supplementary Materials & Methods Fig. 3.

### Microscopy and image analysis

Fluorescent and brightfield images were acquired using a Nikon Eclipse e600 microscope fitted with a Retiga 2000R camera (Q-Imaging, Image Pro premier software). Images were contrasted and analysed using Fiji ImageJ (ImageJ version 1.52e software for Windows (ImageJ Software, National Institutes of Health, USA, available at: http://rsb.info.nih.gov/ij/)). The isotype control acted as the negative reference. All images from one batch of staining were treated equally.

Numbers of Ly6G + cells were counted manually on at least five 10 × FOV per slide. Number of F4/80 + cells were quantified using a macroinstruction on six 20 × FOV per slide. Number of Ly6G + or F4/80 + cells were divided by the necrotic area of the respective slide to give number of cells per necrotic area. Separation of the inner and outer necrotic area is demonstrated in Supplementary Materials & Methods Fig. 5. Percentage area of Sema7a positivity on DAB staining was quantified using a Fiji image J macroinstruction on six 20 × FOV per slide.

Confocal microscopy was performed with an inverted Leica TCS SP8 Confocal microscope. High magnification images used the Nyquist criterion to give the maximum resolution using 60 × objective lens. Images were converted from Z- stacks to Maximum Intensity Projections, and contrast adjusted using Fiji ImageJ.

### TUNEL assay

**Fig. 4:**
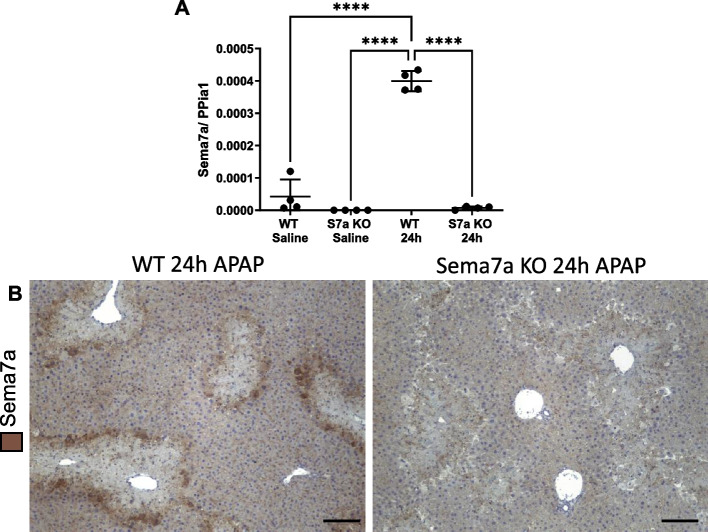
Sema7a KO mice do not express Sema7a Results from WT and Sema7A KO mice 24 h post saline or 350 mg/kg APAP. **A** Hepatic lysate qPCR, Sem7a mRNA expression relative to housekeeper PPIA1. WT APAP-ALI treated mice have significantly elevated expression at 24 h post APAP compared to all other groups (ANOVA, *p* < 0.0001, Tukey’s multiple comparisons, *p* ≤ 0.0001). *n* = 4. **B** Representative hepatic Sema7a (DAB) labelled sections from WT and Sema7a mice 24 h post APAP, showing no expression in WT mice

Terminal deoxynucleotidyl transferase–mediated biotinylated deoxy-uridine triphosphate nickend labelling (TUNEL) assay, was performed on 4 µm FFPE tissue according to manufacturer instructions (In Situ Cell Death Detection Kit, TMR Red, Roche). TUNEL + DAPI + nuclei were imaged and quantified using the Perkin Elmer Operetta high content imaging system and Columbus software (Supplementary Materials & Methods Fig. 4).

### Protein quantification

#### Protein extraction

60 mg liver tissue was homogenised with a Tissue Tearor (Biospec Products) in cold Meso Scale Diagnostics (MSD) lysis buffer (150 mM NaCl, 20 mM Tris pH7.5, 1 mM EGTA, 1 mM EDTA, 1% Triton X-100, 1X Halt Protease inhibitor Cocktail (Thermo Scientific)). Homogenates were slowly mixed for 30 min, then centrifuged for 10 min 20,000 × g at 4 °C. The aqueous supernatant was removed, and the protein concentration was determined using a Pierce BCA Protein Assay Kit (Thermo Scientific).

#### Quantification of cytokines in mouse serum and liver

10 cytokines were multiplexed (IFN-γ, IL-10, IL-12p70, IL-1β, IL-2, IL-4, IL-5, IL-6, CXCL1, TNF) in 25 µL mouse serum using the V-PLEX Pro-inflammatory Panel 1’ mouse plate (MSD), following the manufacturer instructions.

The plate was read on the QuickPlex SQ 120 analyser (MSD). Standard curves were used to quantify the concentration of the respective cytokine.

#### Sema7a enzyme-linked immunosorbent assay (ELISA)

The Mouse LS Bio sandwich ELISA kit was used to quantify Sema7a in mouse liver protein homogenate, diluted to 0.5 mg/ml, or mouse serum, diluted 1:5. Samples were diluted in sample diluent and assayed in duplicate by following the manufacturer’s instructions (Manufacturer; LS-F6958).

### Quantitative reverse transcriptase PCR (qRT-PCR)

40 mg of liver tissue was homogenised in 500 µL Qiazol (Qiagen). Homogenates were mixed with 100 µL chloroform, incubated at RT for 3 min, and then centrifuged at 4 °C 1200 × g, for 15 min. The aqueous supernatant was removed and mixed in an equal volume of 70% ethanol. RNA was extracted using an RNAeasy Kit according to manufacturer instructions (Qiagen). Reverse Transcription and Real Time-qPCR was performed using Qiagen Quantitect and Quantifast reagents on a LightCycler 480 II (Roche). Commercial primers (*peptidylprolyl isomerase A (PPIA)*, *Sema7a*, QT00173488) from Qiagen’s Quantitect range were used. Gene expression was normalised to the housekeeping gene *PPIA.* Samples were run in technical triplicate.

### Statistics

GraphPad Prism 8 Software was used for all statistical analysis. Data are presented as mean ± SD. Each datapoint represents a mouse. Normality was determined by a Shapiro-Wilks test. To test two sample groups a two-tailed unpaired t-test, with a Welch correction applied if required, or Mann Whitney test was used to compare parametric or non-parametric data respectively. Sample size was based on a power calculation where α = 0.05, desired power = 0.8, or from investigator experience.

For further information on flow cytometry, imaging, and other materials and methods, please refer to the CTAT table and supplementary materials and methods.

## Results

### Sema7a is expressed in peri-necrotic hepatocytes during APAP-ALI

Sema7a protein expression, assessed by immunostaining during a time course of APAP-ALI, (Fig. 1A and Supplementary Fig. 1) was not detected in healthy liver tissue or during the early stages of APAP-ALI (0–8 h). As hepatic necrosis and inflammation progressed from 12 to 24 h post APAP-ALI, Sema7a expression was localised to peri-necrotic cells, with peak expression at 24 h post-APAP-ALI. Sema7a expression diminished as necrosis resolved (36—60 h post APAP-ALI) (Fig. 1B, C).

Since peak Sema7a expression occurs at 24 h post-APAP, we further characterised Sema7a expression at this time point. Sema7a/Hnf4α dual staining identified that peri-necrotic hepatocytes express Sema7a (Fig. 2A). In perfused whole liver, *Sema7a* mRNA expression significantly increased with APAP-ALI compared to healthy mice (p = 0.0053, Fig. 2B). However, hepatic Sema7a protein levels did not significantly change (Fig. 2C). This total hepatic difference between transcribed and translated quantification may reflect release of the protein into circulation. Circulating Sema7a significantly increased in mouse serum at 12 and 24 h post-APAP (*p* = 0.0127 and *p* = 0.007, respectively) (Fig. 2D), and circulating Sema7a correlated with a serum marker of hepatocellular injury, alanine aminotransferase (ALT) (Pearson coefficient (r) = 0.728, Fig. 2E).

As Sema7a is localised to peri-necrotic hepatocytes, we ensured Sema7a does not label dying cells. Sema7a + hepatocytes are negative for both active caspase 3 and TUNEL + DAPI + nuclei (Supplementary Fig. 1) demonstrated by serial stained sections. Therefore, Sema7a is expressed by the viable hepatocytes, which surround necrosis.

### Sema7a expressing hepatocytes directly contact cells expressing Sema7a receptors

The predominant Sema7a receptors are Integrin β1 and Plexin C1. Integrin β1 is ubiquitously expressed across the liver [45] and Plexin C1 is expressed by hepatic stellate cells (HSCs), (Supplementary Fig. 2). Integrin β1 is upregulated by HSCs during fibrosis and Sema7a binding is known to contribute to TGF-beta mediated fibrosis [16]. Sema7a also effects spiny stellate cell function [46], but the impact of APAP-ALI on Sema7a receptor expression and their proximity to Sema7a expressing hepatocytes is unknown. To assess this relationship, we completed a dual stain for Sema7a during APAP-ALI. Integrin β1 + and Plexin C1 + HSCs were detected between the Sema7a + hepatocytes at peak Sema7a expression, suggesting a localised signalling interaction (Fig. 3A&B). At 24 h post APAP-ALI, hepatic Integrin β1 mRNA expression and Plexin C1 protein expression increased compared to healthy mice (Supplementary Fig. 3). In Sema7a KO mice, Plexin C1 but not Integrin β1 expression was reduced at 24 h post APAP (Supplementary Fig. 4, p = 0.0208), suggesting Sema7a promotes the expression of Plexin C1.

### Sema7a KO mice have more liver injury during APAP-ALI

**Fig. 5:**
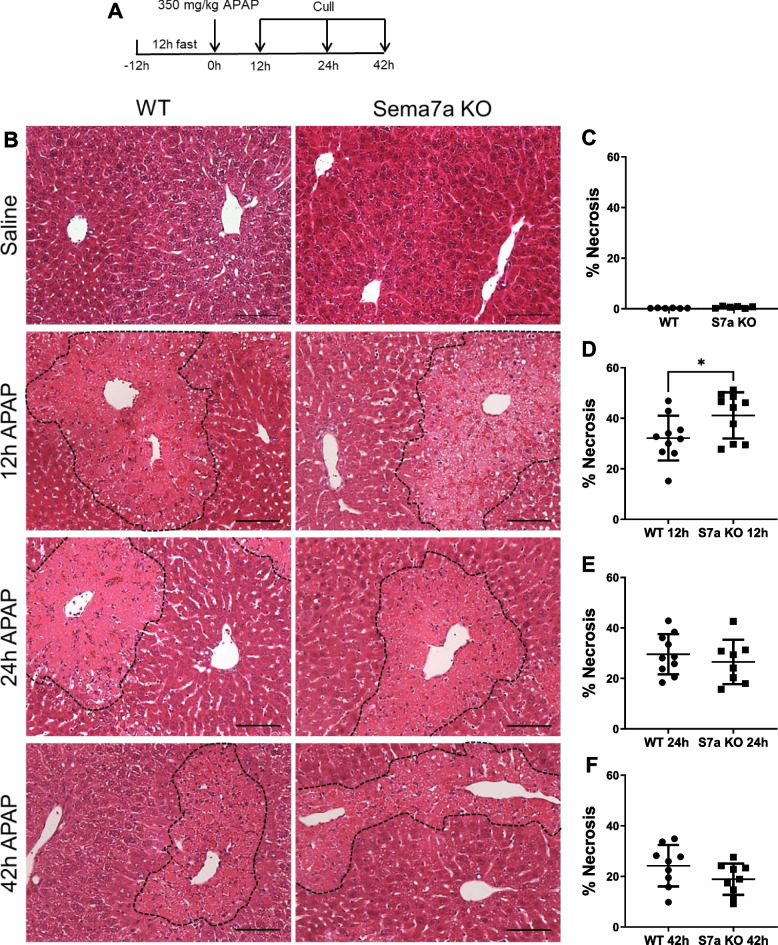
Sema7a KO mice have more liver necrosis during APAP-ALI. **A** Experimental schematic; WT and Sema7a KO mice treated with 350 mg/kg APAP, assessed at 12, 24 and 42 h. **B** Representative liver histological stains from Sema7a WT, and Sema7a KO mice. Necrotic areas outlined with black dashed line. Scale bars 100 µm. **C**-**F** Necrosis quantification, necrotic areas were segmented and quantified using inForm 2.4 (Perkin Elmer) software using inform tissue training for consistent changes; enlarged cell organelles, membrane rupture and cell lysis at 0 h (**C**) 12 h, t-test (*p* = 0.0391) (**D**), 24 h (**E**) and 42 h (**F**) post 350 mg/kg APAP injection. Each datapoint represents a mouse. * *p* < 0.05; ** *p* < 0.01, n ≥ 8 mice/group

To investigation of the importance of peri-necrotic hepatocyte Sema7a expression in APAP-ALI we completed experiments in Sema7a KO mice. We first confirmed deficiency of Sema7a expression in Sema7a KO mice both with hepatic qPCR analysis and immunohistochemistry at 24 h post APAP-ALI (Fig. 4). We also compared LFTs, and liver histology (Fig. 5A-C. & Supplementary Fig. 5) of healthy WT and Sema7a KO mice and showed no significant differences compared to WT mice (Fig. 5A-C. & Supplementary Fig. 5).

**Fig. 6:**
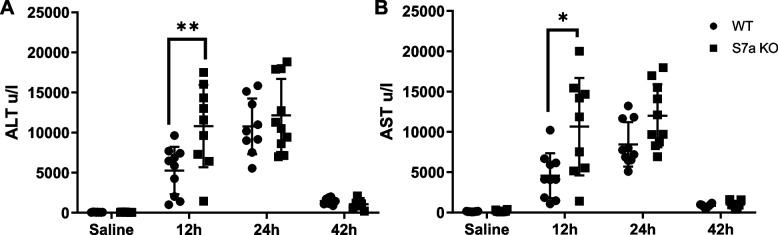
Sema7a KO mice have more liver damage during APAP-ALI. Serum markers of liver injury (LFTs), ALT (**A**) and AST (**B**) were higher at 12 h post 350 mg/kg APAP in Sema7a KO mice compared to WT mice during the time course of APAP-ALI, (t-test, Welch’s correction, *p* = 0.0095 and *p* = 0.0107 respectively). Each datapoint represents a mouse. * *p* < 0.05; ** *p* < 0.01, n ≥ 7 mice/group. During recovery, at 42 h post APAP-ALI, Sema7a KO mice and Sema7a WT mice exhibited similar necrosis (Fig. 5F) and proliferation (Supplementary Fig. 6). This suggests Sema7a does not directly influence recovery from APAP-ALI. However, Sema7a KO mice had raised ALP at this time point (*p* = 0.0306, Supplementary Fig. 5)

Comparing Sema7a KO and WT mice during a time course of 350 mg/kg APAP-ALI identified that Sema7a KO mice exhibit significantly more hepatic necrosis at 12 h post APAP-ALI (Fig. 5D-F,p = 0.0391). This was supported by higher serum ALT (p = 0.0095) and aspartate aminotransferase (AST) (p = 0.0107, Fig. 6A&B) at 12 h post APAP-ALI. Although increased necrosis was not evident at 24 h post APAP-ALI and only a trended increase was present in LFTs, Sema7a KO mice had higher serum bilirubin at 24 h, which can increase with reduced liver function, (p = 0.0109, Supplementary Fig. 5).

### Absence of Sema7a on peri-necrotic hepatocytes results in diffuse cell death during APAP-ALI

**Fig. 7:**
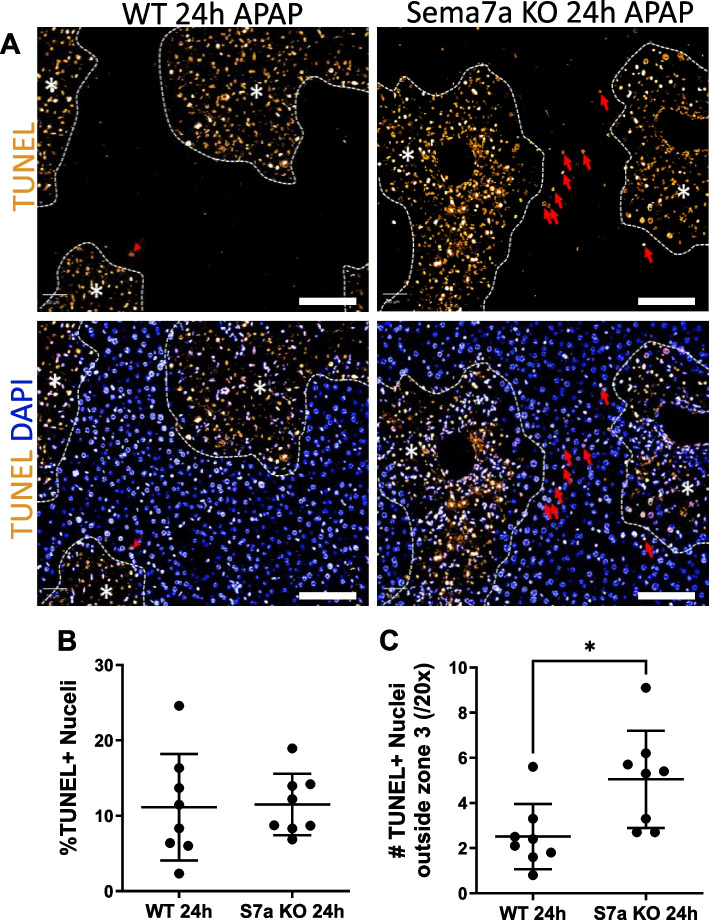
Absence of Sema7a on peri-necrotic hepatocytes results in diffuse cell death during APAP-ALI. **A** TUNEL assay (yellow), DAPI (blue), in Sema7a WT and Sema7a KO mice 24 h post APAP-ALI. Necrotic areas outlined with white lines and *. Red arrows are TUNEL + nuclei outside the necrotic area. **B** Percentage of TUNEL + DAPI + nuclei per FOV. **C** Number of TUNEL + DAPI + nuclei outside the necrotic area, per FOV (t-test, p0.0151). FOV, fields of view. Scale bars 100 µm. Each datapoint represents a mouse. *n* = 8 mice/group. *p < 0.05, **p < 0.01

Alongside the documented elevated hepatocellular injury and necrosis, deficiency of Sema7a in peri-necrotic hepatocytes was associated with alterations in cell death distribution. At 24 h post APAP-ALI, during peak Sema7a expression, Sema7a KO mice had the same percentage of TUNEL + nuclei as WT mice, but TUNEL + nuclei were not contained within the centrilobular necrotic areas and there were TUNEL + cells in the surrounding healthy parenchyma (Fig. 7, *p* = 0.0151), suggesting Sema7a plays a localised role to limit the extent of liver tissue damage at 24 h post APAP-ALI.

**Fig. 8:**
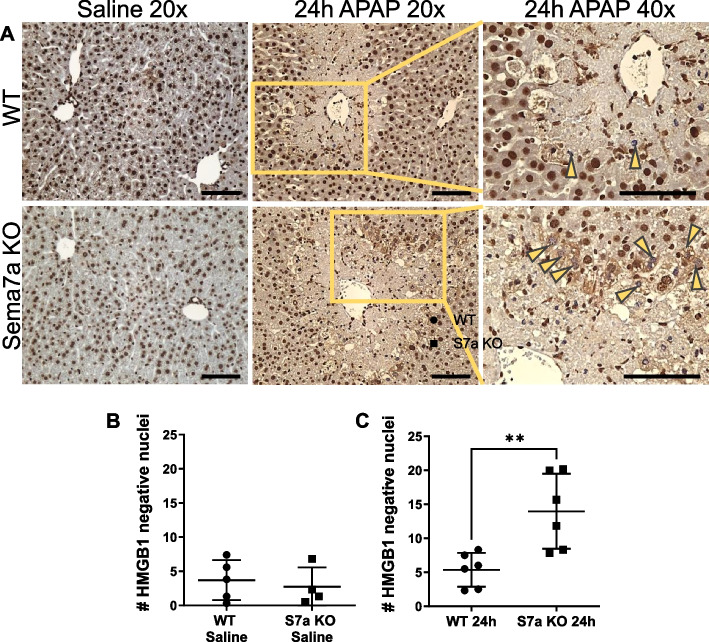
Absence of Sema7a on peri-necrotic hepatocytes results in higher cell stress during APAP-ALI. Results from WT and Sema7a KO mice 24 h post saline or 350 mg/kg APAP. **A** Representative HMGB1 (DAB) labelled hepatic sections from Sema7a WT (top) and Sema7a KO (bottom) mice with indicated treatments. Inset, area for 40x. Yellow arrowheads, show HMGB1 negative nuclei. **B** Average number of HMGB1- nuclei per FOV quantified in healthy mice. **C** Average number of HMGB1- nuclei per FOV following APAP-ALI were higher in Sema7a KO mice (t-test, *p* = 0.0058). FOV, fields of view. Scale bars 100 µm. Each datapoint represents a mouse. *n* ≥ 4 mice/group, **p < 0.01

To further assess if Sema7a KO mice have more local tissue inflammation and cell stress during APAP-ALI, High Mobility Group Box 1 (HMGB1) localisation was examined. In healthy mice, HMGB1 is retained in the nucleus, but is released from necrotic cells, during injury, therefore non-nuclear or cytosolic HMGB1 is a sign of cell stress [47]. Healthy Sema7a WT and Sema7a KO mice displayed similar nuclear HMGB1 staining (Fig. 8). However, at 24 h post APAP-ALI, Sema7a KO mice had significantly more peri-necrotic hepatocytes with HMGB1 negative nuclei (*p* = 0.0058, Fig. 8).

### Sema7a KO mice have more inflammation during APAP-ALI

**Fig. 9:**
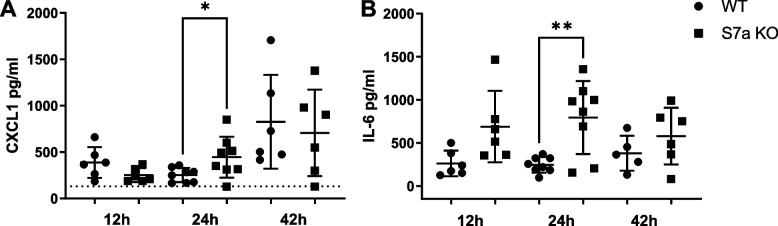
Sema7a KO mice have increased serum pro-inflammatory cytokines. Serum cytokines in Sema7a WT and Sema7a KO mice following 350 mg/kg APAP. **A** CXCL1, a neutrophil chemoattractant, was higher in Sema7a KO mice serum at 24 h (t-test, Welch’s correction, *p* = 0.0459). **B** IL-6 was higher in Sema7a KO mice serum at 24 h (t-test, Welch’s correction, *p* = 0.0078)

Inflammation is known to contribute to APAP induced injury, and Sema7a is known as an immunomodulator, so to start assessing the effect of Sema7a deficiency on inflammation during APAP-ALI, a panel of pro-inflammatory cytokines were quantified in the serum of Sema7a WT and Sema7a KO mice throughout the time course. Sema7a KO mice had more CXCL1 (*p* = 0.0459) and IL-6 (*p* = 0.0078) at 24 h post APAP-ALI (Fig. 9), indicative of a higher systemic pro-inflammatory response.

### Sema7a KO mice have more neutrophils within necrosis during APAP-ALI

**Fig. 10:**
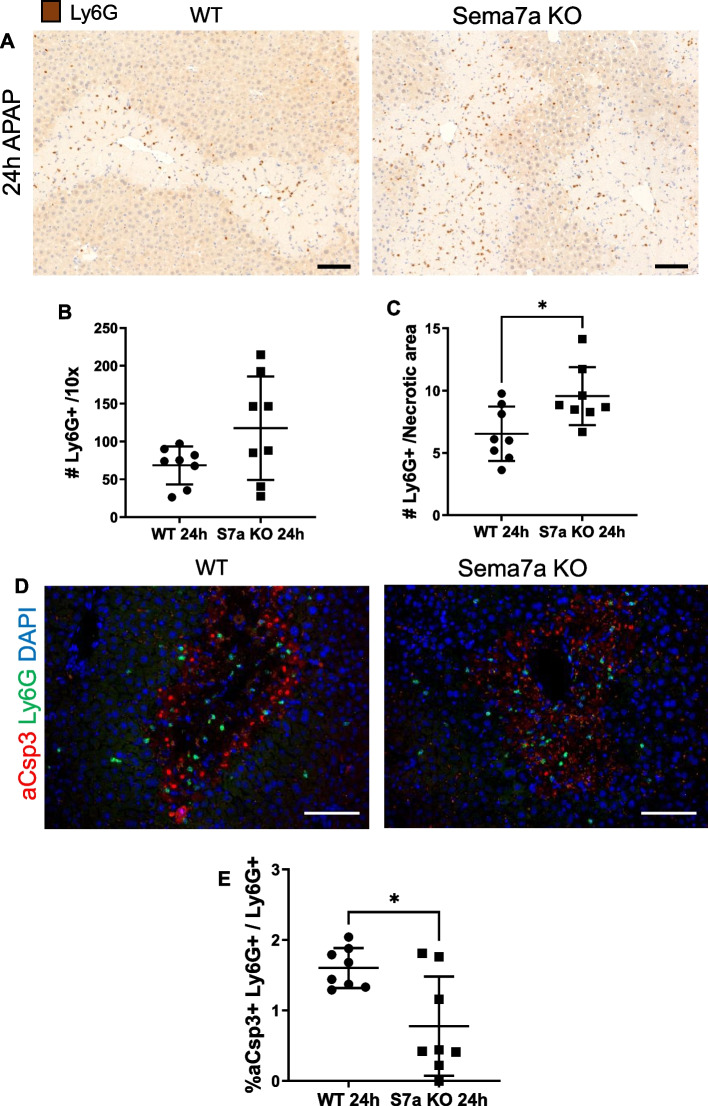
Sema7a KO mice have increased neutrophils infiltrating necrotic areas. Results from Sema7a WT and Sema7a KO mice 24 h post 350 mg/kg APAP. **A** Representative Ly6G labelled hepatic sections in Sema7a WT (left) and Sema7a KO mice (right). **B** Average number of Ly6G neutrophils per FOV. **C** The average number of neutrophils within necrosis was higher in Sema7a KO mice (t-test, *p* = 0.0182). **E** Ly6G (green) and active Caspase 3 (aCsp3, red) expression in WT (centre) and Sema7a KO mice (right) at 24 h post-APAP-ALI. Quantified (right) as percentage of apoptotic neutrophils (aCsp3 + , Ly6G +) in the total Ly6G population. Unpaired t test with Welch's correction, *p* = 0.0129. Scale bars 100 µm. Each datapoint represents a mouse. Unpaired t-test, unless stated. *p < 0.05, **p < 0.01

HMGB1 and CXCL1 are known neutrophil chemo-attractants. We therefore quantified numbers of Ly6G + neutrophils in liver tissue using both immunohistochemistry (Fig. 10 A-B) and flow cytometry, and in whole blood by flow cytometry. Sema7a WT and Sema7a KO mice had the same total number of hepatic and circulating neutrophils, throughout the APAP-ALI time course (Fig. 10 A-B, Supplementary Fig. 7–9). However, Sema7a KO mice had a greater density of neutrophils within centrilobular necrotic areas at 24 h post APAP-ALI, when Sema7a expression was otherwise highest in WT mice (Fig. 10 C; *p* = 0.0182). WT mice hepatic neutrophils do not express Sema7a, but express both known receptors for Sema7a, Plexin C1 and Integrin αvβ1 (Supplementary Fig. 10).

Neutrophils rapidly migrate into liver after APAP-ALI [48] and we recently identified hepatic neutrophil cleaved caspase 3 expression is markedly reduced, consistent with increased activation and survival [49]. At 24 h post APAP-ALI, we show only 1.6% of neutrophils expressed active caspase 3 in Sema7a WT mice consistent with this finding, and this was significantly lower in Sema7a KO mice (0.7%, Fig. 10D-E, *p* = 0.0129), indicating less neutrophil apoptosis. Although this reduction is small, we believe this is significant in this already activated cell population. Further reduction in this marker of cell death could indicate increased neutrophil activation in the Sema7a KO mice or altered phagocytosis/clearance of apoptotic neutrophils. Macrophages remove apoptotic neutrophils by phagocytosis [50–53] but this has yet to be shown in vivo after APAP-ALI. We investigated the effect of Sema7a on phagocytosis with an in vivo PKH assay during APAP-ALI and found no differences between Sema7a KO and WT mice for monocytes, macrophages, or neutrophils (Supplementary Fig. 11). Therefore, Sema7a does not directly impact this form of phagocytosis, though leucocytosis clearance is mediated by Sema7a in other models [25].

### Sema7a KO mice have less F4/80 + macrophages during APAP-ALI

**Fig. 11:**
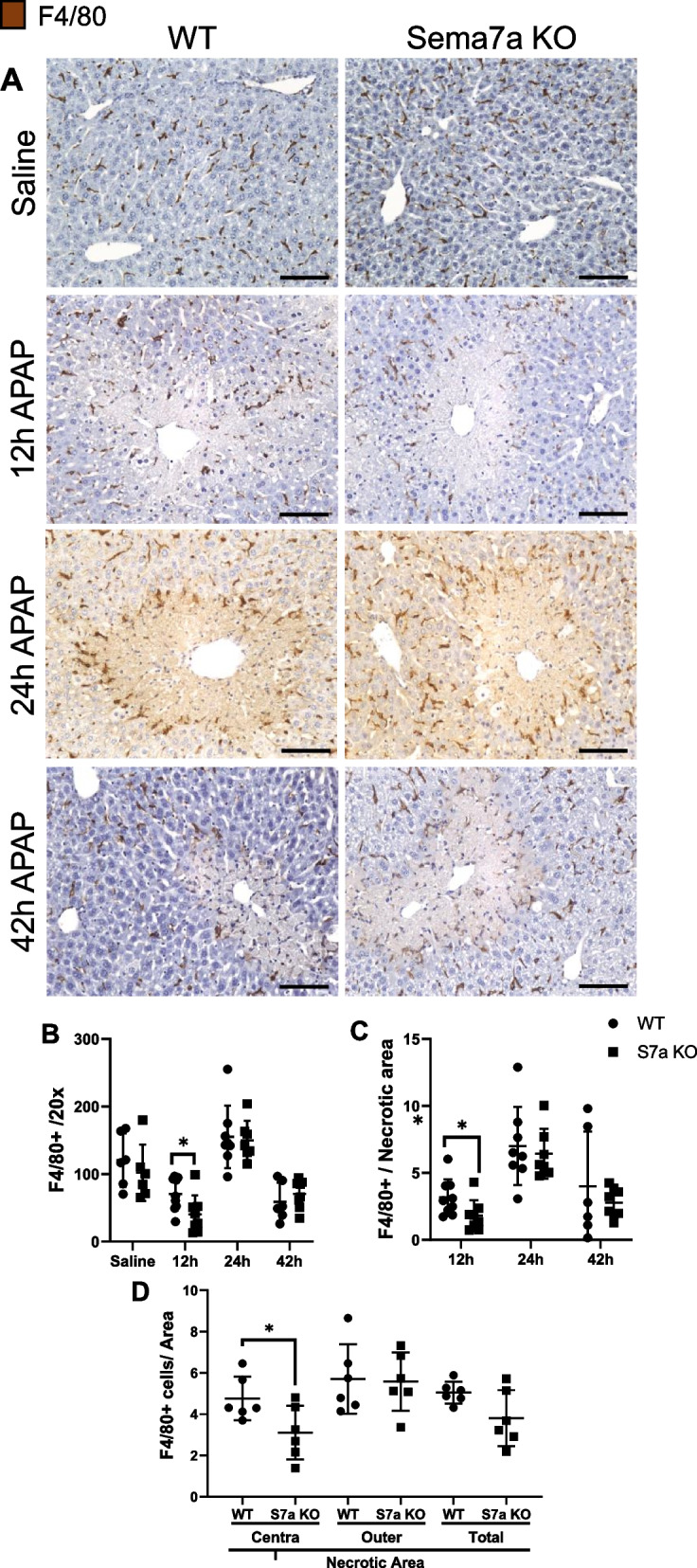
Sema7a KO mice show less F4/80 + macrophages at 12 h APAP-ALI, with aberrant localisation. Results are from WT and Sema7a KO mice receiving 350 mg/kg APAP or saline. **A** Representative images of liver F4/80 + macrophages in WT (left) and Sema7a KO mice (right) at the indicated timepoints. **B** Average number of F4/80 + macrophages per FOV, (12 h t-test, *p* = 0.0315). **C** Average number of F4/80 + macrophages in necrotic areas during the time course of APAP-ALI (12 h t-test, *p* = 0.044). **D** Location of F4/80 + macrophages in the necrotic area at 24 h APAP-ALI. The necrotic area was separated into outer and central necrotic zones. Average number of F4/80 + macrophages per necrotic zones was calculated in Sema7a WT and Sema7a KO mice (12 h t-test, *p* = 0.0366). Scale bars 100 μm. Each datapoint represents an individual mouse. *n* ≥ 6 mice/group. *p < 0.05

To examine if a Sema7a deficiency impacted liver macrophages, a F4/80 stain was performed (Fig. 11A). Healthy Sema7a WT and Sema7a KO mice have comparable numbers of F4/80 + macrophages. However, at 12 h post APAP-ALI, total hepatic F4/80 + macrophages numbers were significantly lower in Sema7a KO mice (*p* = 0.0315, Fig. 11B), with a reduced density within the necrotic area (p = 0.044, Fig. 11C) indicating a possible exaggeration of the previously recognised KC depletion [30, 32].

From 12 to 24 h post APAP-ALI the hepatic F4/80 + population increased in both Sema7a WT and Sema7a mice, consistent with previous literature of infiltrating macrophages replenishing the liver macrophage pool [32]. At 24 h post APAP-ALI total hepatic macrophages were not different (Fig. 11B Supplementary Fig. 12), however, Sema7a KO mice had less central necrosis macrophages (*p* = 0.0366, Fig. 11D), suggesting Sema7a has a localised role which facilitates F4/80 + macrophage migration into the necrotic area. During recovery, total hepatic F4/80 + macrophages numbers declined similarly in both Sema7a WT and Sema7a KO mice (Fig. 11B).

## Discussion

Novel strategies to limit the extent of necrosis, or to promote healthy liver repair from APAP-ALI are urgently required for APAP-overdose patients [2, 5]. Understanding APAP-ALI and secondary inflammation are crucial to identify potential targets for patient therapy. Here, we report a novel role of the immunomodulator Sema7a during APAP-ALI.

Sema7a is expressed by hepatocytes, shown here and previously [54]. It’s highly localised, and increased expression by peri–necrotic hepatocytes during APAP-ALI is a novel finding. Serum concentrations of Sema7a correlate with serum injury biomarkers as injury progresses, consistent with the findings in human paediatric patients with acute inflammatory abdominal disease [26]. Sema7a deficiency in this APAP-ALI model was detrimental, leading to increased hepatocellular damage, demonstrated by raised LFTs, more non-nuclear HMGB1 cells, higher necrosis, and TUNEL positivity in liver parenchyma. This finding is in contrast to various non-hepatic models, where Sema7a promotes tissue injury and fibrosis [15, 55, 56]. This protective finding is also different to chronic hepatic injury models, such as CCl_4_-induced liver injury, where Sema7a promotes hepatic fibrosis and Il6 and Ccl2 expression [16]. More recent hepatobiliary focussed publications identified mutations in Sema7a causing a gain of function, progressing disease; non-alcoholic fatty liver disease (NAFLD) and intrahepatic cholestasis [54, 57]. Paradoxical to these studies, in acute sepsis and peritonitis, a protective role of Sema7a has been identified through immunomodulation [25, 26]. Here we show a protective action of Sema7 in ALI that has not previously been recognised.

We identified that Plexin C1 was reduced in Sema7a KO mice, at 24 h APAP-ALI (Supplementary Fig. 6), suggesting Sema7a promotes Plexin C1 expression on HSCs. This increased expression of receptor Plexin C1 has also been identified in paediatric systemic vasculitis [58]. As both receptors are present on neutrophils this difference may influence their migration and activation.

Alongside higher hepatic injury and altered receptor expression, Sema7a KO mice had more inflammation during APAP-ALI with higher CXCL1 and IL-6 at 24 h. This finding also coincided with alterations in inflammatory cell populations, with both reduced central necrotic zone F4/80 + macrophages and higher neutrophils. As Sema7a is a macrophage chemoattractant [20], its lack of expression on peri-necrotic hepatocytes may have reduced monocyte derived macrophage migration, which are known to replenish depleted F4/80 macrophages after in APAP-ALI [32, 59].

As well as hepatic necrosis macrophage reduction we identified that Sema7a KO resulted in higher necrosis neutrophils. Neutrophils are attracted to the liver during injury by DAMPs, such as passively released HMGB1 [42–44] or cytokines such as CXCL1 [60]. In Sema7a KO mice, the hepatic necrotic area neutrophil increase may be due to the documented HMGB1 and CXCL1 elevations increasing local neutrophil migration or retention. Alternatively, increased numbers may be due to increased survival and potentially activation during APAP-induced inflammation [61]. Körner et al. identified Sema7a dampened neutrophil recruitment and was crucial for inflammation resolution, including leukocyte clearance in murine peritonitis. Furthermore, Sema7a deficiency may also delay neutrophil migration out of the necrotic tissue, as endothelial Sema7a has been shown to facilitate neutrophil transmigration during pulmonary injury, [18, 29]. Further interrogations of how Sema7a impacts in vivo neutrophil migration in APAP-ALI are warranted. Sema7a promotes extracellular matrix remodelling factors including *Cathepsin S* [56] and neutrophils secrete cathepsins to degrade basement membranes in sterile thermal liver injury, enabling them to exit the damaged tissue [62]. The increased numbers of necrotic area neutrophils may be one of the reasons for higher hepatic necrosis, consistent with a pro-inflammatory role postulated by several authors [21, 42].

The higher hepatic damage in the Sema7a KO mice and higher inflammation in this study may also by due to altered immune cell function. Macrophages are highly plastic cells which acquire distinct phenotypes depending on molecular cues in their microenvironment [32, 33] and Sema7a is a recognised cue of macrophage and monocyte activity [15, 63]. We did not find evidence of deficiency of innate immune cell PKH phagocytosis, so reduced efferocytosis of hepatic neutrophils, an important component of inflammation resolution [35, 64], is unlikely in APAP-ALI, despite this finding in peritonitis [26]. Therefore, the increased neutrophils in the necrotic area at 24 h APAP-ALI in Sema7a KO mice could be a result of reduced macrophage migration [35, 65]. Sema7a has been shown as crucial for the resolution of severe inflammation, through polarization of macrophages to a pro-resolving phenotype in acute peritonitis [26]. This would be an important future direction for research of Sema7a in acute liver injuries, given the knowledge that restorative Ly6C^lo^ macrophages have been shown to be crucial for appropriate liver repair via phagocytosis [35, 36] and secretion of IL-10 to reduce inflammation [28].

## Conclusion

In conclusion, we have identified a novel hepatoprotective role of Sema7a in acute liver injury, with associated innate immunity changes, highlighting a potential immunomodulatory role behind this phenotype. Hepatic Sema7a expression increased during the injury phase of APAP-ALI and peak expression was localised to the peri-necrotic area. Without peri-necrotic Sema7a there was more hepatic necrosis during injury (12 h) and cellular damage (TUNEL + and HMGB1-) was not restricted the necrotic region. Sema7a deficiency resulted in more inflammation at 24 h post APAP-ALI, with increased IL6 and CXCL1 quantification. There were more neutrophils within necrosis, with lower cleaved caspase 3, indicating increased activation. There were also lower necrotic area macrophages, key cells for liver repair following APAP-ALI. Future work to further clarify the mechanisms behind the hepatoprotective effect of Sema7a in APAP-ALI are warranted. Better understanding of these mechanisms might identify this as a novel therapeutic target to limit the spread of necrosis and reduce inflammation during acute tissue injury.

## Supplementary Information

Supplementary Material 1.

## Data Availability

No datasets were generated or analysed during the current study.

## Abbreviations

APAP: Acetaminophen
APAP-ALI: Acetaminophen-induced liver injury
ALF: Acute liver failure
ALT: Alanine aminotransferase
APAP-ALI: APAP-induced acute liver injury
AST: Aspartate aminotransferase
CXCL1: Chemokine (C-X-C motif) ligand 1
DAMPs: Danger-associated-molecular patterns
HSCs: Hepatic stellate cells
HMGB1: High mobility group box 1
IL: Interleukin
KCs: Kupffer cells
KO: Knockout
LFTs: Liver function tests
NAC: N-acetylcysteine
NAFLD: Non-alcoholic fatty liver disease
Sema7a: Semaphorin 7a
WT: Wild-type
