# Supplementary material for: Semaphorin 7a is protective through immune modulation during acetaminophen-induced liver injury
Source: J Inflamm (Lond). 2025 Mar 20;22:13. doi: 10.1186/s12950-025-00429-x (PMC11927371; doi:10.1186/s12950-025-00429-x)
Supplement: Supplementary file 1 — Supplementary Material 1. [file 12950_2025_429_MOESM1_ESM.pdf]

Semaphorin 7a reduces hepatic macrophage depletion and acetaminophen induced liver injury

Eilidh J. Livingstone [1], Jennifer Ann Cartwright [2], Lara Campana [1,2], Philip Starkey Lewis [1,3], Benjamin J. Dwyer [1], Rhona Aird [1], Tak Yung Man [1], Matthieu Vermeren [1], Adriano Giorgio Rossi [2], Luke Boulter [4], Stuart John Forbes [1,3]

Table of Contents

Supplementary data 5

Supplementary Fig. 1 Sema7a+ cells are viable and surround the necrosis 6

Supplementary Fig. 2 Plexin C1 is expressed by hepatic stellate cells 8

Supplementary Fig. 3 Plexin C1 and Integrin β1 expression during APAP-ALI 9

Supplementary Fig. 4 Effect of Sema7a deficiency on Integrin β1 and PlexinC1 expression during APAP-ALI 10

Supplementary Fig. 5 Sema7a KO mice have higher LFTs during APAP-ALI 11

Supplementary Fig. 6 Sema7a KO and WT mice have similar proliferation at 42 hours APAP-ALI 13

Supplementary Fig. 7 Neutrophils infiltrate the liver during APAP-ALI 15

Supplementary Fig. 8 Neutrophil frequency in the liver of at 24 hours post APAP-ALI 16

Supplementary Fig. 9 Frequency of circulating leukocytes at 12 and 24 hours post APAP-ALI 18

Supplementary Fig. 10 Neutrophils express Sema7a receptors 20

Supplementary Fig. 12 WT and Sema7a KO mice have similar frequencies of macrophages and monocytes in the liver at 24 hours post APAP-ALI 22

Supplementary Fig. 11 Hepatic immune cell phagocytosis is similar between WT and Sema7a KO mice 24

Supplementary materials and methods 25

Antibodies used and Isotype controls 25

Supplementary Table 1 Primary antibodies used for immunohistochemistry and immunofluorescence........25

Supplementary Table 2 Secondary antibodies used for immunohistochemistry and immunofluorescence........27

Supplementary Materials & Methods Fig. 1 Isotype controls for Sema7a the APAP time course experiment........28

Supplementary Materials & Methods Fig. 2 Controls for immunofluorescent stains.....29

Supplementary Materials & Methods Fig. 3 Isotype controls for DAB stains........31

Image Analysis........32

Quantification of necrotic area........32

Supplementary Materials & Methods Fig. 4 Necrosis analysis using the inForm software........32

Supplementary Materials & Methods Fig. 5 Strategy to quantify TUNEL+ DAPI+ cells........33

Supplementary Materials & Methods Fig. 6 Quantification of the number of F4/80+ cells per area........34

Flow cytometry........35

Flow cytometry on peripheral blood........35

Sema7a receptor expression on neutrophils........35

Supplementary Table 3 Sema7a receptor panel on peripheral blood........36

Numbers of neutrophils and monocytes in peripheral blood........37

Supplementary Table 4 Neutrophil and monocyte panel for peripheral blood........37

In vivo phagocytosis flow cytometry analysis of blood..38
Supplementary Table 5 In vivo phagocytosis flow cytometry panel for blood................38
Liver digestion and non-parenchymal cell isolation and staining for flow cytometry.........39
Calculating the absolute number of NPCs per gram of liver..39
NPC staining for flow cytometry and in vivo phagocytosis 40
Supplementary Table 6 In vivo phagocytosis flow cytometry panel for NPCs of the liver 41
References..41

Supplementary data

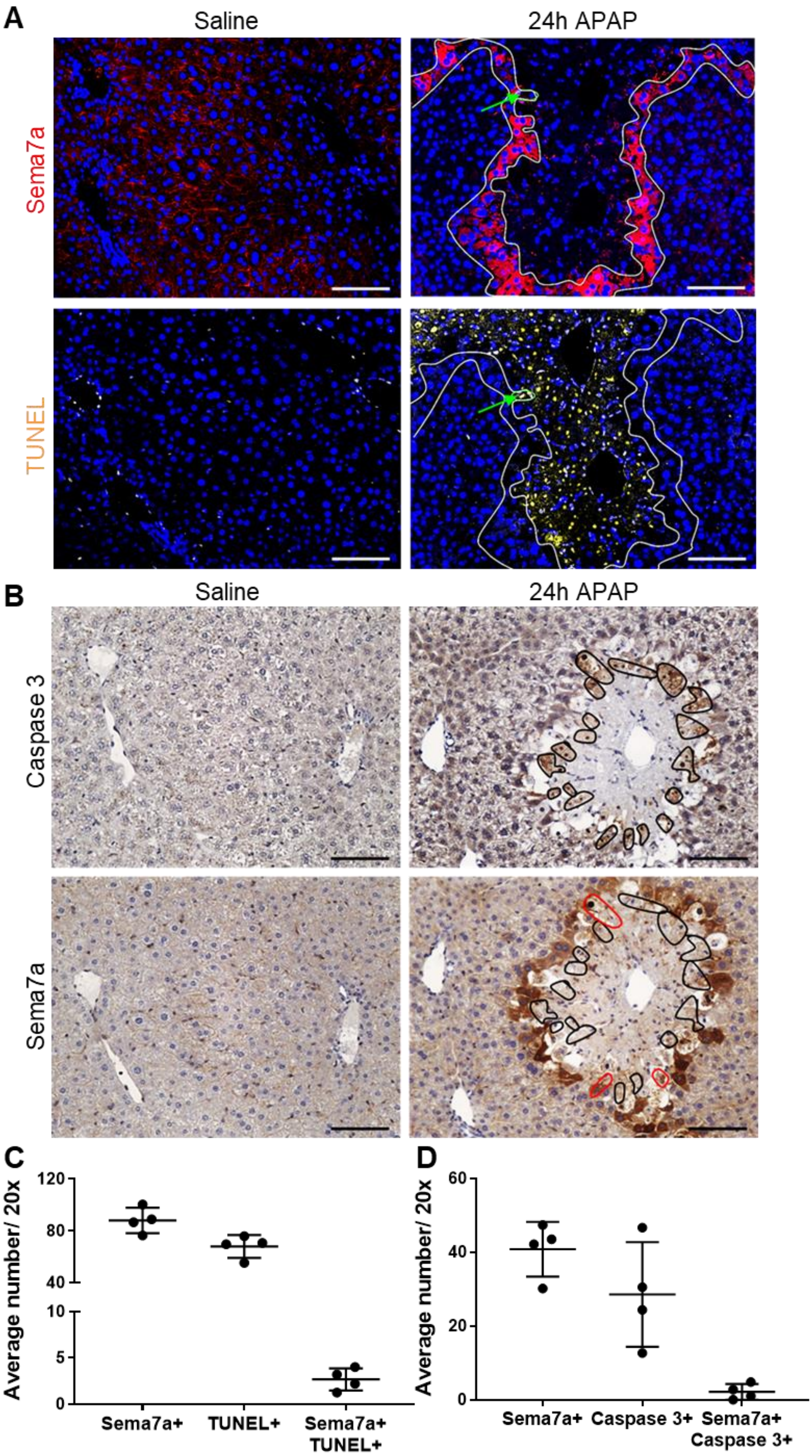

Supplementary Fig. 1 Sema7a+ cells are viable and surround the necrosis

(A) Serial sections of Sema7a (top) and a TUNEL assay (bottom) in mice treated with 24 hours saline (left) or 350 mg/kg APAP (right). White outline, Sema7a+ region. Green arrows, cells dual positive for TUNEL and Sema7a (Sema7a+ TUNEL+)

(B) Serial staining of Caspase 3 (top) and Sema7a (bottom) 24 hours post saline (left) or APAP (right). Black outline, Caspase 3+ cells. Red, cells dual positive for Caspase 3 and Sema7a (Sema7a+ Caspase 3+).

(C) Number of Sema7a+, TUNEL+ or dual positive Sema7a+, TUNEL+ cells at 24 hours post 350 mg/kg APAP treatment.

(D) Average number of Sema7a+, Caspase 3+ or dual positive cells at 24 hours post 350mg/kg APAP treatment.

Each datapoint represents a mouse. Scale bars 100μm.

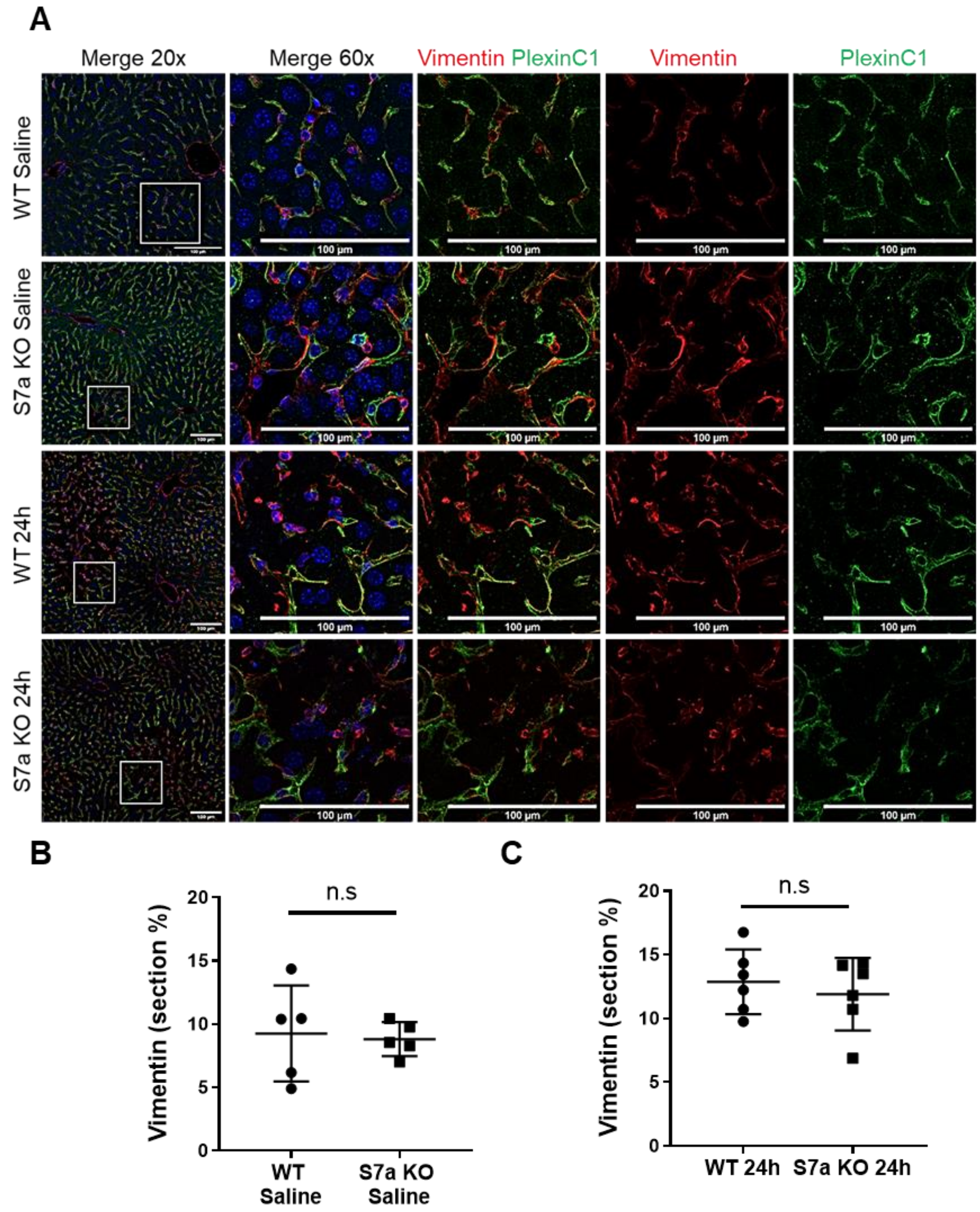
A
Merge 20x
Merge 60x
Vimentin PlexinC1
Vimentin
PlexinC1
WT Saline
S7a KO Saline
WT 24h
S7a KO 24h
100 µm
B
n.s
Vimentin (section %)
20
15
10
5
0
WT Saline
S7a KO Saline
C
n.s
Vimentin (section %)
20
15
10
5
0
WT 24h
S7a KO 24h

Supplementary Fig. 2 Plexin C1 is expressed by hepatic stellate cells

(A) Confocal images of Vimentin (red, HSC marker), and Plexin C1 (green) with DAPI (blue) in WT and Sema7a KO mice treated with saline (top two rows) or 24 hours APAP (bottom two rows). Left column shows a representative 20x image. White inset, area used for 60x image on the right.

(B) Average percentage area of vimentin expression in saline treated mice

(C) Average percentage area of vimentin expression in WT and Sema7a KO mice treated at 24 hours post APAP APAP-ALI

Scale bars 100 µm. Unpaired t-test.

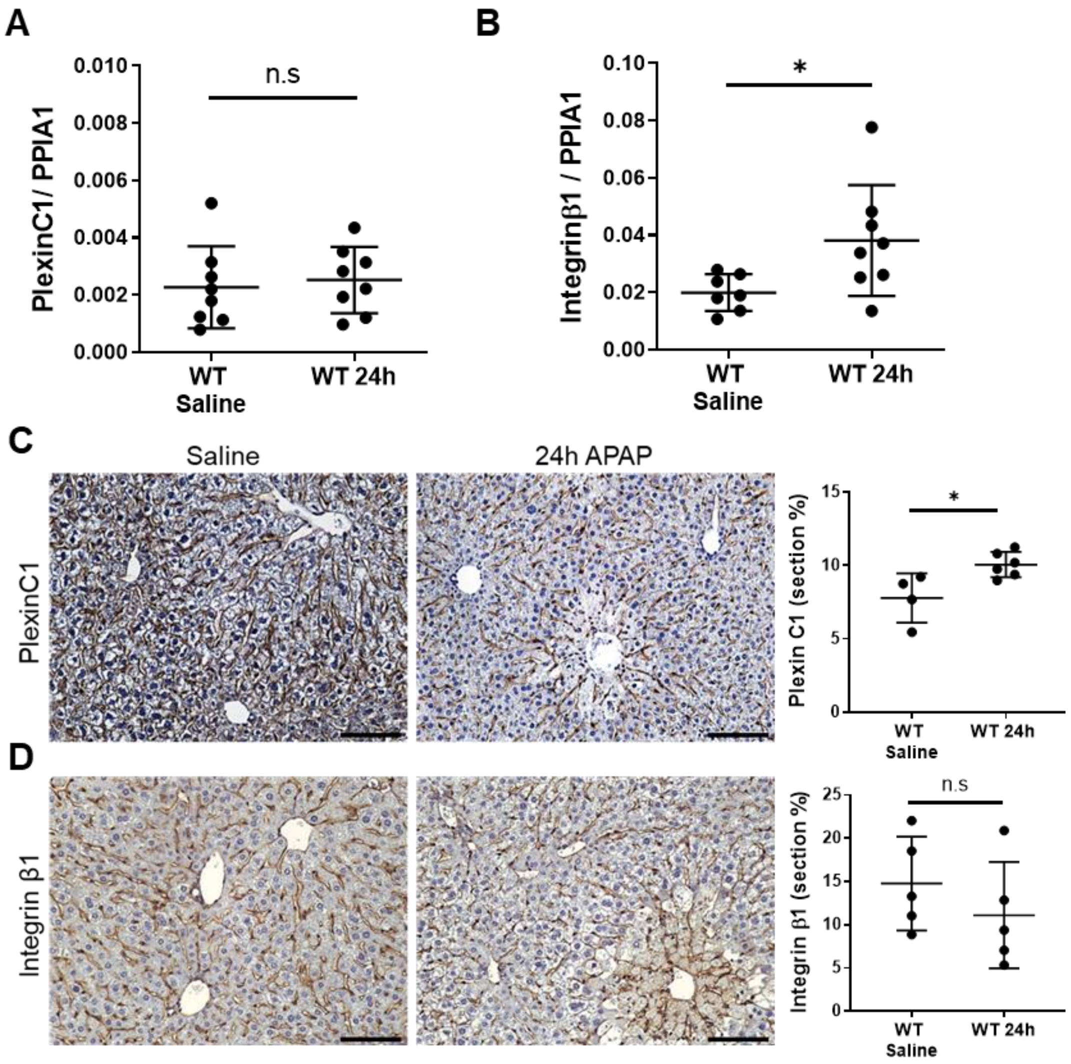

Supplementary Fig. 3 Plexin C1 and Integrin β1 expression during APAP-ALI

Expression of the Sema7a receptors, Plexin C1 and Integrin β1 were examined in WT mice which were treated with saline (left), or 24 hours post APAP (right).

(A) *PlexinC1* mRNA expression, relative to *PPIA1* in whole liver lysate.

(B) *Integrin β1* mRNA expression, relative to *PPIA1* in whole liver lysate (Welch's correction applied).

(C) Plexin C1 IHC. Area of Plexin C1 positivity is quantified (right)

(D) Integrin β1 IHC. Area of Integrin β1 positivity is quantified (right)

Scale bars 100 µm. *p<0.05; Unpaired t-test.

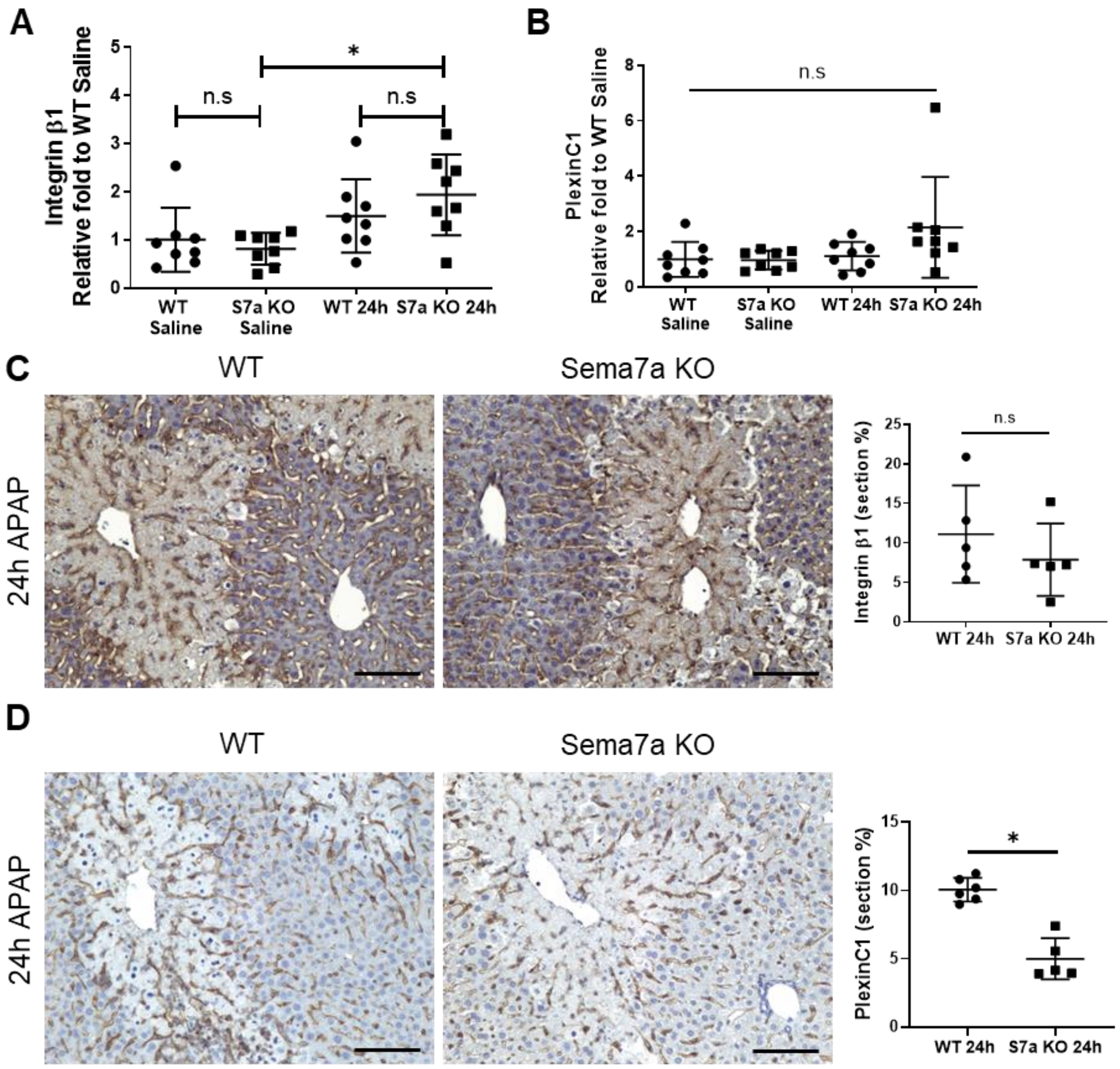

Supplementary Fig. 4 Effect of Sema7a deficiency on Integrin β1 and PlexinC1 expression during APAP-ALI

(A) *Integrin β1* and (B) *PlexinC1* mRNA expression at 24 hours saline or APAP treatment in WT and Sema7a KO mice, relative to WT saline treated mice.

Kruskal-Wallis test, Dunn's multiple comparison test. Sema7a KO saline vs, Sema7a KO 24 hours post APAP p=0.0427

(C) Integrin β1 and (D) Plexin C1 staining in WT (left) and Sema7a KO (right) mice at 24 hours post APAP-ALI treatment. Average area of staining positivity is quantified right. Unpaired t-test. *p<0.05. Scale bars 100 µm.

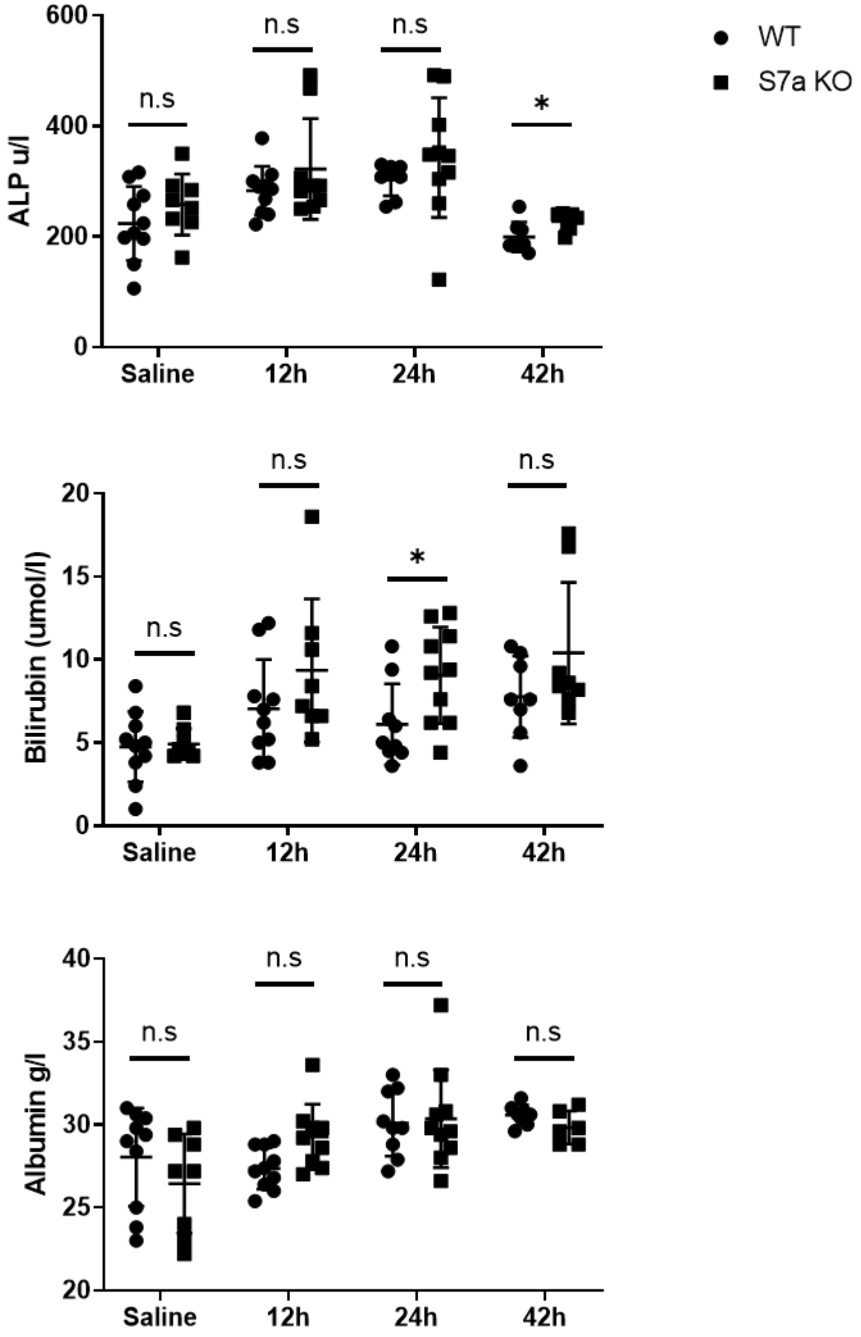

Supplementary Fig. 5 Sema7a KO mice have higher LFTs during APAP-ALI

Time course of serum LFTs from WT and Sema7a KO mice treated with 350mg/kg APAP. Alkaline phosphatase (ALP), bilirubin and albumin.

Unpaired t-test. *p<0.05

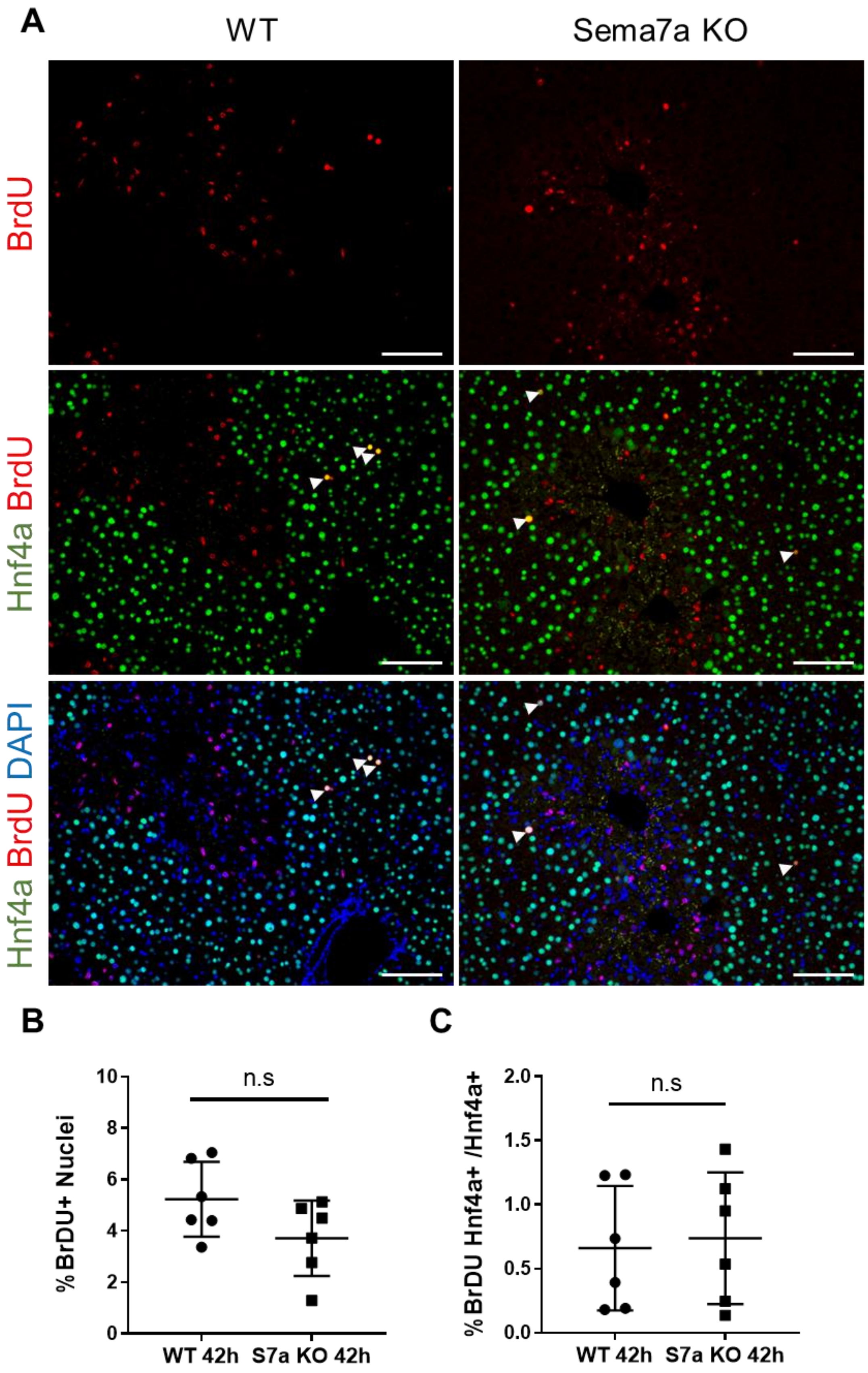
A
WT
Sema7a KO
BrdU
Hnf4a BrdU
Hnf4a BrdU DAPI
B
C
n.s
% BrDU+ Nuclei
10
8
6
4
2
0
WT 42h
S7a KO 42h
n.s
% BrDU Hnf4a+ /Hnf4a+
2.0
1.5
1.0
0.5
0.0
WT 42h
S7a KO 42h

Supplementary Fig. 6 Sema7a KO and WT mice have similar proliferation at 42 hours APAP-ALI

WT and Sema7a KO mice were injected with 100uL of 1mg/ml BrdU before being humanely sacrificed.

(A) BrdU incorporation (red) and Hnf4a (green), dual positive yellow, in WT (left) and Sema7a KO (right) mice at 42 hours post APAP injection. White arrow heads indicate proliferating hepatocytes.

(B) Percentage of BrdU+ nuclei in WT and Sema7a KO mice

(C) Percentage of dual positive Hnf4α and BrdU hepatocytes

Images were quantified using the Columbus software. Scale bars 100μm.

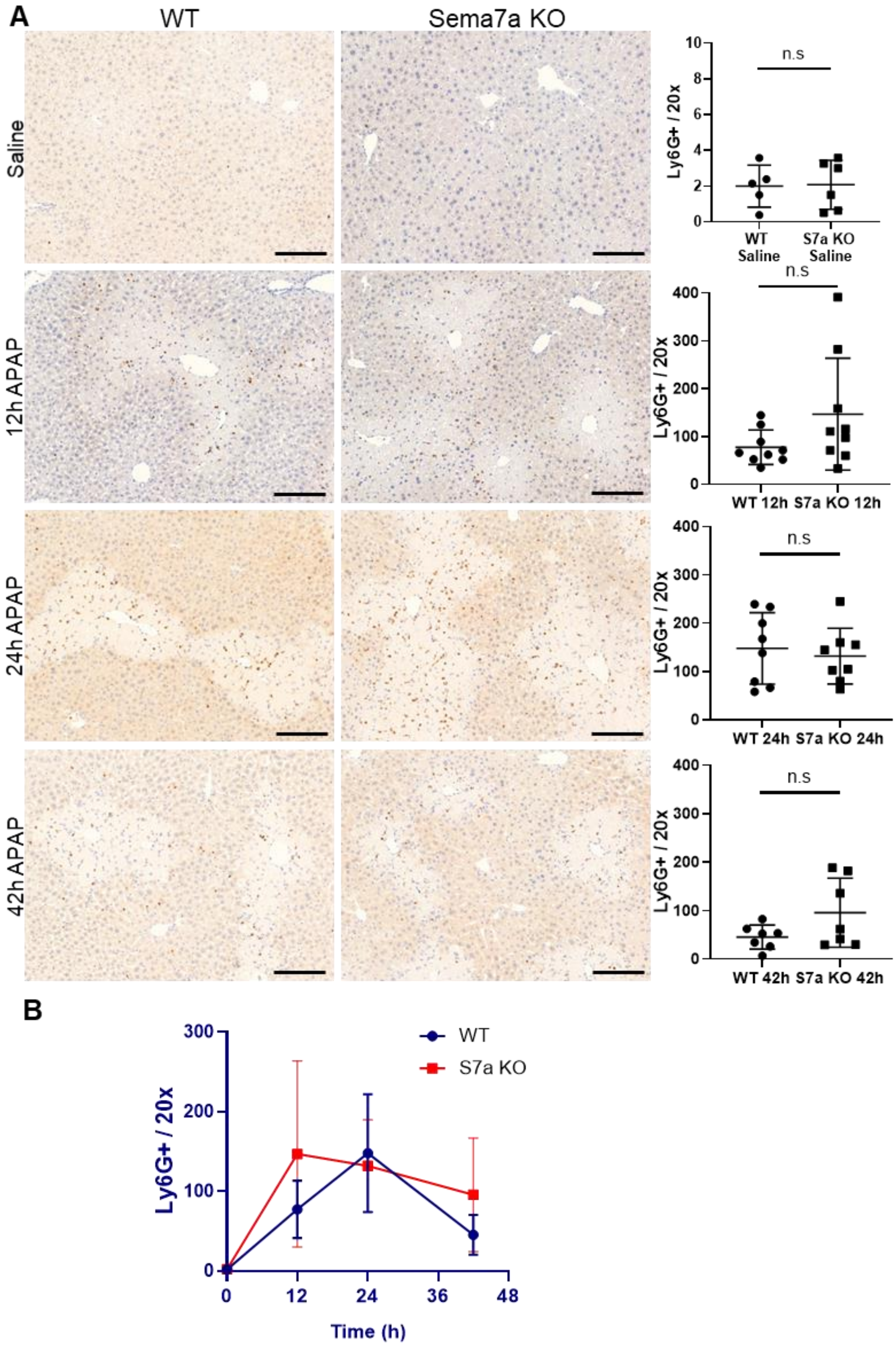
A
WT
Sema7a KO
Saline
12h APAP
24h APAP
42h APAP
Ly6G+ / 20x
n.s
WT Saline
S7a KO Saline
WT 12h
S7a KO 12h
WT 24h
S7a KO 24h
WT 42h
S7a KO 42h
B
WT
S7a KO
Time (h)

Supplementary Fig. 7 Neutrophils infiltrate the liver during APAP-ALI

(A) Ly6G staining for neutrophils in WT (left) and Sema7a KO mice (centre) over a time course of APAP. Number of Ly6G+ neutrophils per 20x FOV are quantified (right). In saline treated (top) or APAP treated mice at 12 (second row), 24 (third row) and 42 (bottom row) hours post APAP-ALI. Unpaired t-test. 12 and 24 hours required a Welch test.

(B) Average number of F4/80+ macrophages per 20x FOV, during the APAP-ALI time course. Two-way ANOVA, n.s.

WT mice, blue. Sema7a KO mice, red. Scale bars 100µm. *$p < 0.05$.

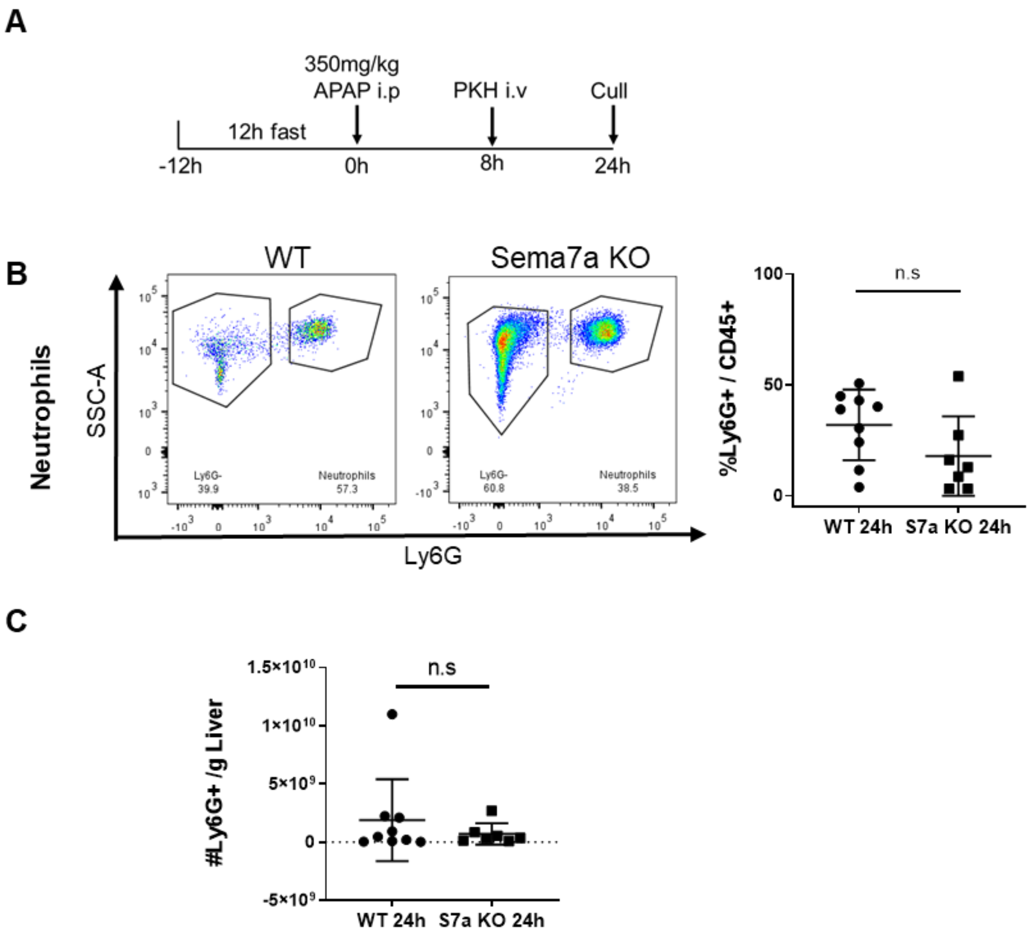

Supplementary Fig. 8 Neutrophil frequency in the liver of at 24 hours post APAP-ALI

(A) Schematic of experiment.

(B) Non parenchymal cells (NPCs) were isolated from the livers of WT (left) and Sema7a KO mice (centre). Quantification (right) shows the frequency of the neutrophil population (Live, Lineage -, CD45+ Ly6G+). Unpaired t-test

(C) Absolute count of neutrophils, per gram of liver in WT and Sema7a KO mice at 24 hours post APAP-ALI

Mann Whitney test

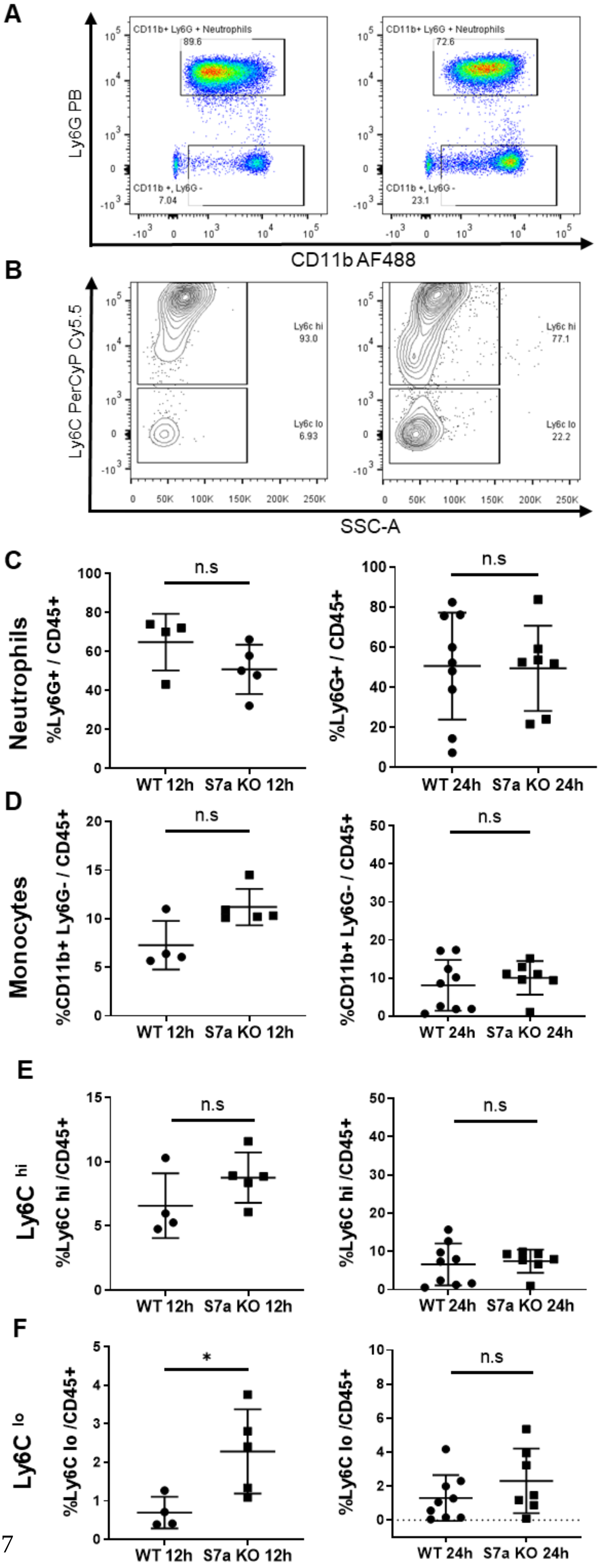
A
Ly6G PB
CD11b AF488
CD11b+ Ly6G + Neutrophils
89.6
72.6
CD11b +, Ly6G -
7.04
23.1
B
Ly6C PerCyP Cy5.5
SSC-A
Ly6c hi
93.0
77.1
Ly6c lo
6.93
22.2
C
Neutrophils
%Ly6G+ / CD45+
n.s
WT 12h
S7a KO 12h
WT 24h
S7a KO 24h
D
Monocytes
%CD11b+ Ly6G- / CD45+
E
Ly6C hi
%Ly6C hi /CD45+
F
Ly6C lo
%Ly6C lo /CD45+
*

Supplementary Fig. 9 Frequency of circulating leukocytes at 12 and 24 hours post APAP-ALI

Peripheral blood was collected from WT (left) and Sema7a KO mice (right) at 12 and 24 hours post 350mg/kg APAP. Flow cytometry plots show the gating strategy to identify populations of:

(A) Neutrophils and,

(B) Ly6C^{hi} and Ly6C^{lo} Monocytes

The Neutrophil (C), Monocytes (D), Ly6C^{hi} monocytes (Mann Whitney test) (E), and Ly6C^{lo} monocytes (F) populations were quantified as a percentage of the live, CD45+ population at both the 12 hours (left) and 24 hours (right) post APAP-ALI time points.

Data from three experiments is shown. Unpaired t-test, unless otherwise stated.

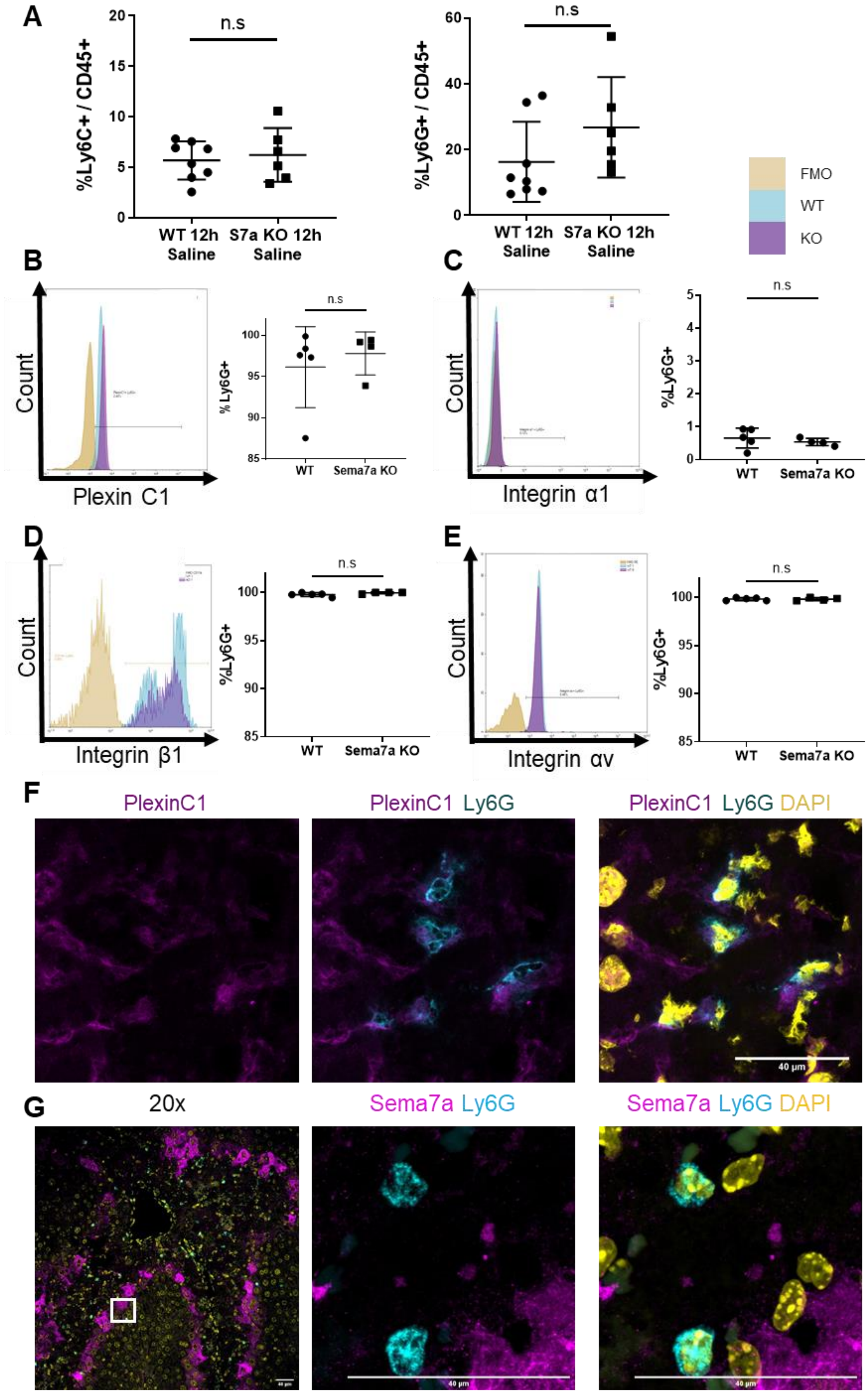
A
%Ly6C+ / CD45+
n.s
WT 12h Saline
S7a KO 12h Saline
%Ly6G+ / CD45+
n.s
FMO
WT
KO
B
Count
Plexin C1
% Ly6G+
n.s
WT
Sema7a KO
C
Count
Integrin α1
%Ly6G+
n.s
WT
Sema7a KO
D
Count
Integrin β1
%Ly6G+
n.s
WT
Sema7a KO
E
Count
Integrin αv
%Ly6G+
n.s
WT
Sema7a KO
F
PlexinC1
PlexinC1 Ly6G
PlexinC1 Ly6G DAPI
40 µm
G
20x
Sema7a Ly6G
Sema7a Ly6G DAPI
40 µm

Supplementary Fig. 10 Neutrophils express Sema7a receptors

A) Flow cytometry analysis of Ly6C+ monocytes (left) and Ly6G+ neutrophils (right) in the peripheral blood of WT and Sema7a KO mice 12 hours after saline treatment.

Sema7a receptor expression on neutrophils isolated from untreated WT (cyan) and Sema7a KO (magenta) mice: (B) PlexinC1, (C) Integrin α1, (D) Integrin B1 and (E) Integrin αv. Quantified (right). FMO control (yellow). Each datapoint represents a mouse.

Confocal imaging of neutrophils Ly6G (cyan) with: (F) Plexin C1 (magenta); or (G) Sema7a (magenta). White inset, area for 60x.

Images from WT mice 24 hours APAP-ALI. DAPI counterstain (yellow). Scale bars 40 µm

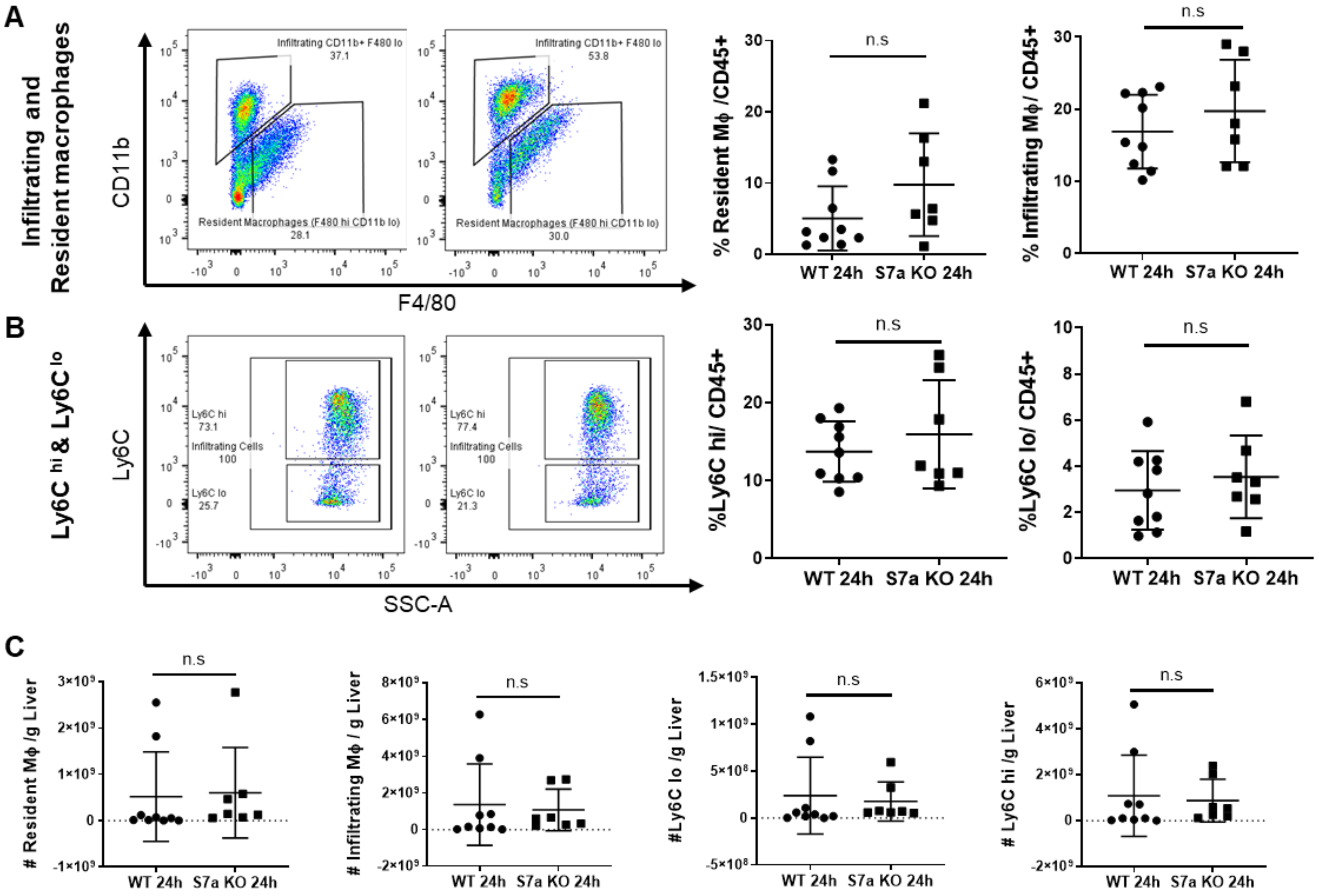
A
Infiltrating and Resident macrophages
CD11b
F4/80
Infiltrating CD11b+ F480 lo
37.1
Resident Macrophages (F480 hi CD11b lo)
28.1
Infiltrating CD11b+ F480 lo
53.8
Resident Macrophages (F480 hi CD11b lo)
30.0
% Resident Mϕ /CD45+
% Infiltrating Mϕ/ CD45+
n.s
WT 24h
S7a KO 24h
B
Ly6C hi & Ly6C lo
Ly6C
SSC-A
Ly6C hi
73.1
Infiltrating Cells
100
Ly6C lo
25.7
Ly6C hi
77.4
Infiltrating Cells
100
Ly6C lo
21.3
%Ly6C hi/ CD45+
%Ly6C lo/ CD45+
C
Resident Mϕ /g Liver
Infiltrating Mϕ / g Liver
#Ly6C lo /g Liver
Ly6C hi /g Liver

Supplementary Fig. 11 WT and Sema7a KO mice have similar frequencies of macrophages and monocytes in the liver at 24 hours post APAP-ALI

Non parenchymal cells (NPCs) were isolated from the livers of WT (left) and Sema7a KO mice (centre). Quantification shows the frequency of NPC populations in the Live, Lineage -, CD45+ population:

(A) Resident macrophages (quantified left, Mann Whitney test); Infiltrating macrophages (quantified right). Unpaired t-test.

(B) Infiltrating macrophages were examined for $Ly6C^{hi}$ and $Ly6C^{lo}$ expression. Unpaired t-test

(C) Absolute counts of resident macrophages, infiltrating macrophages, $Ly6C^{hi}$ monocytes, and $Ly6C^{lo}$ macrophages per gram of liver in WT and Sema7a KO mice at 24 hours post APAP-ALI. Mann Whitney test.

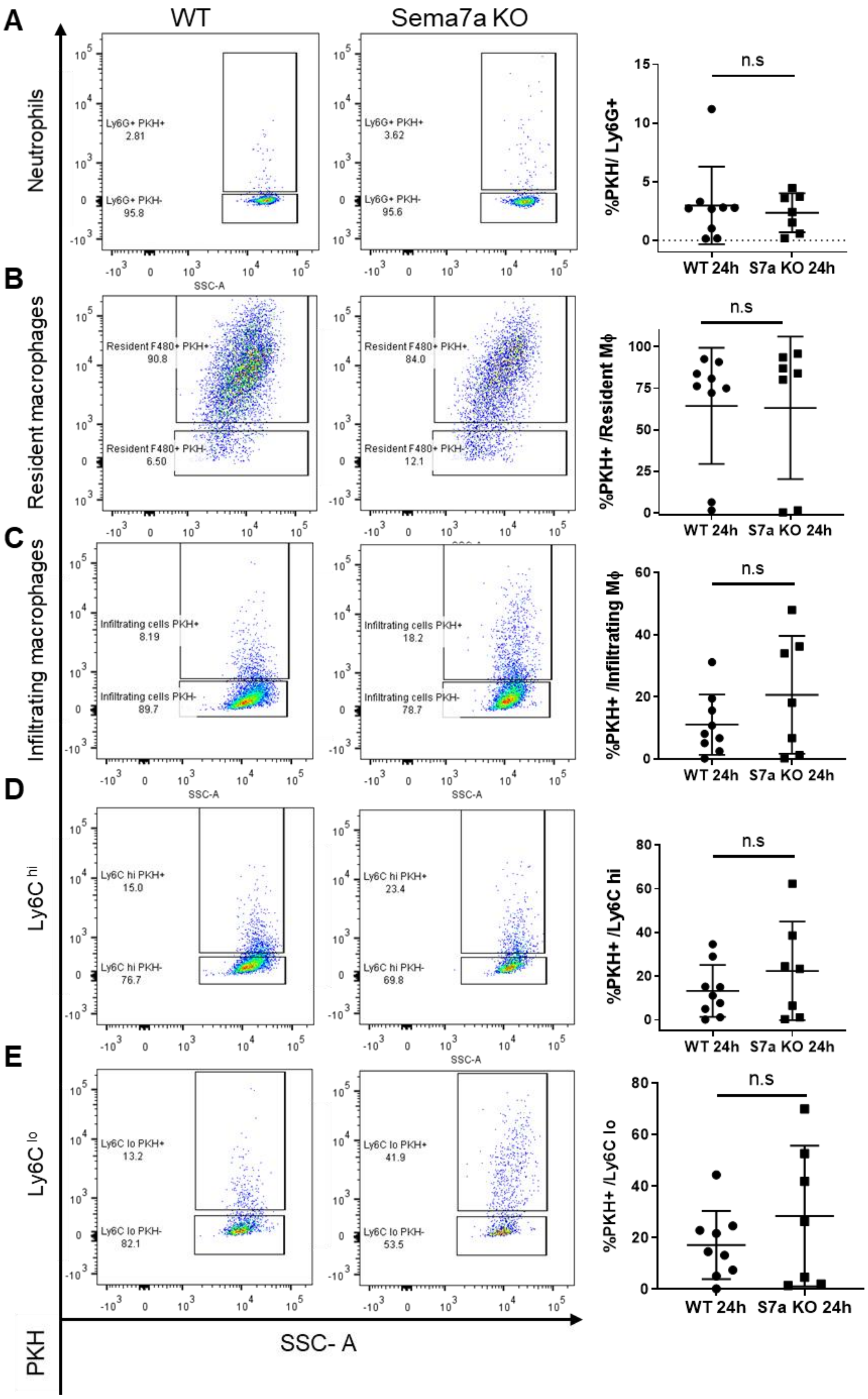

A
B
C
D
E
WT
Sema7a KO
Neutrophils
Resident macrophages
Infiltrating macrophages
Ly6C hi
Ly6C lo
PKH
SSC- A
Ly6G+ PKH+ 2.81
Ly6G+ PKH- 95.8
Ly6G+ PKH+ 3.62
Ly6G+ PKH- 95.6
Resident F480+ PKH+ 90.8
Resident F480+ PKH- 6.50
Resident F480+ PKH+ 84.0
Resident F480+ PKH- 12.1
Infiltrating cells PKH+ 8.19
Infiltrating cells PKH- 89.7
Infiltrating cells PKH+ 18.2
Infiltrating cells PKH- 78.7
Ly6C hi PKH+ 15.0
Ly6C hi PKH- 76.7
Ly6C hi PKH+ 23.4
Ly6C hi PKH- 69.8
Ly6C lo PKH+ 13.2
Ly6C lo PKH- 82.1
Ly6C lo PKH+ 41.9
Ly6C lo PKH- 53.5
SSC-A
%PKH/ Ly6G+
%PKH+ /Resident Mϕ
%PKH+ /Infiltrating Mϕ
%PKH+ /Ly6C hi
%PKH+ /Ly6C lo
n.s
WT 24h
S7a KO 24h

Supplementary Fig. 12 Hepatic immune cell phagocytosis is similar between WT and Sema7a KO mice

(A) *In vivo* phagocytosis by liver NPCs isolated from WT (left) and Sema7a KO mice (centre) at 24 hours post APAP injection. Graphs (right) show the frequency of phagocytosis cells in each NPC population:

(B) Neutrophils (Mann Whitney test),

(C) Resident macrophages (Mann Whitney test),

Infiltrating macrophages, which was separated into (D) $Ly6C^{hi}$ and (E) $Ly6C^{lo}$ infiltrating macrophages

Data from three experiments is shown. Unpaired t-test, unless stated. Each datapoint represents a mouse.

Supplementary materials and methods

Antibodies used and Isotype controls

Supplementary Table 1 Primary antibodies used for immunohistochemistry and immunofluorescence

Antibody	Dilution from stock	Fixation	Antigen retrieval	Manufacturer	Cat. Number	Lot number	Host
Active Caspase 3	1:200	Formalin	15min Na C	BD Pharmingen	BD559565		Rb
Cyp2e1	1:500	Formalin	15min Na C	Atlas antibodies	HPA009128		Rb
F4/80	1:200	Methanol : Acetone	N/A	Abcam	ab6640		Rt
HMGB1	1:500	Formalin	15min Na C	Abcam	ab18256		Rb
Hnf4a	1:200	Formalin	15min TE	Perseus Proteomics Inc.	PP-H1415-00		Ms
Integrin β1	1:200	Methanol : Acetone	N/A	Millipore	MAB1997		Rt
Ly6G	1:500	Formalin	15min TE	Biolegend	127601		Rt
Plexin C1	1:150	Formalin	15min TE	R&D Systems	AF5375		Shp

Sema7a	1:100	Formalin	15min<br>TE	Abcam	ab23578	GR306<br>146-2	Rb
Vimentin	1:500	Formalin	15min<br>TE	Abcam	ab92547		Rb

Na C, Sodium Citrate; TE, Tris ETDA pH 8; Ms, Mouse; Rb, Rabbit; Rt, Rat; Shp, Sheep.

Supplementary Table 2 Secondary antibodies used for immunohistochemistry and immunofluorescence

Target species	Conjugate	Fluorophore	Dilution from stock	Manufacturer	Cat. Number	Host
Mouse	Fluorescent	Alexa 488	1:200	Invitrogen	A21202	Dk
Rabbit	Biotinylated	-	1:200	Vector	BA-1000	Gt
Rabbit	Fluorescent	Alexa 555	1:200	Invitrogen	A31572	Dk
Rat	Biotinylated	-	1:200	Vector	BA-9400	Gt
Rat	Fluorescent	Alexa 488	1:200	Invitrogen	A21208	Dk
Sheep	Biotinylated	-	1:200	Vector	BA-6000	Rb
Sheep	Fluorescent	Alexa 555	1:200	Invitrogen	A21436	Dk
Sheep	Fluorescent	Alexa 488	1:200	Invitrogen	A11015	Dk

Dk, Donkey; Gt, Goat; Rb, Rabbit;

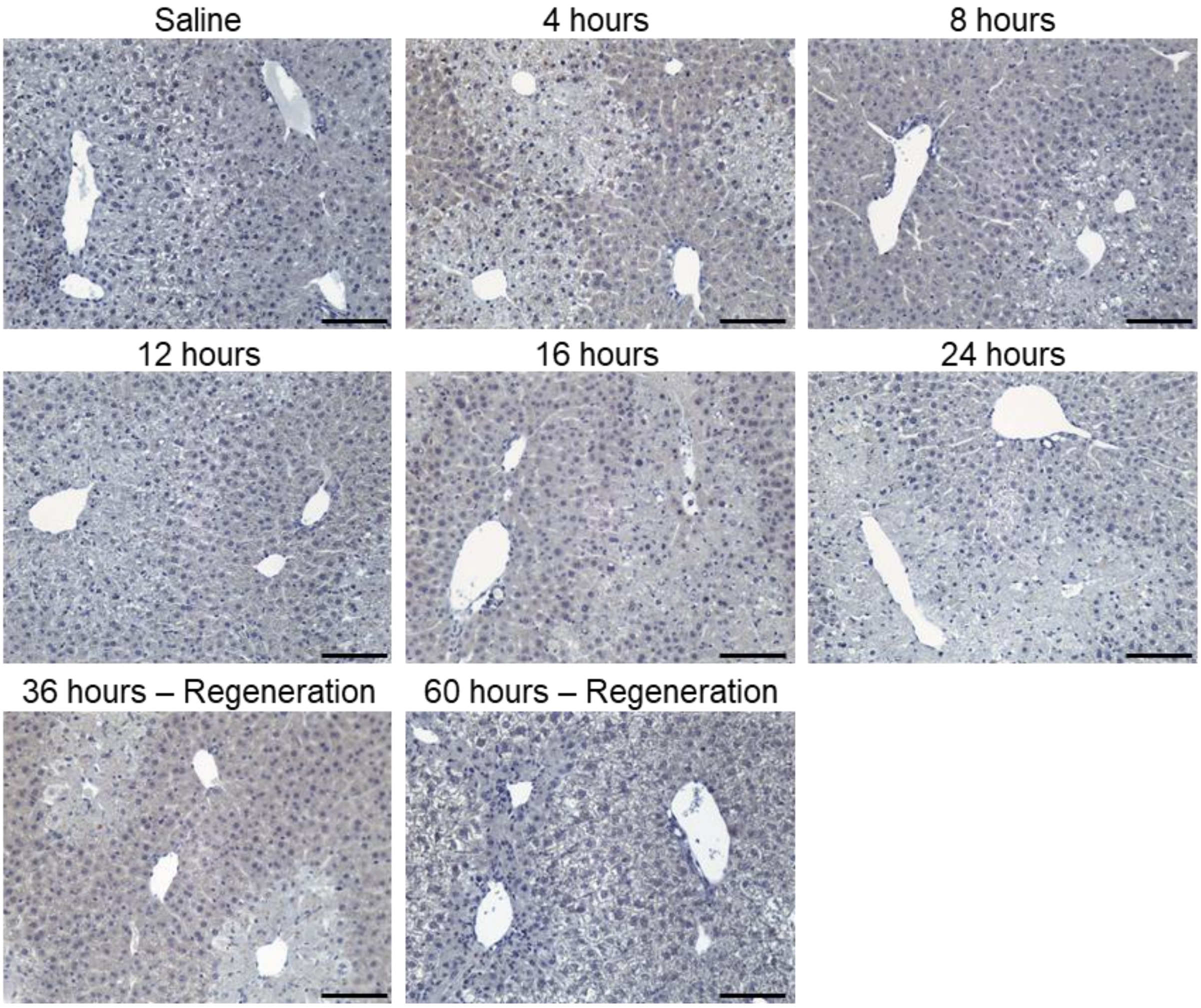

Supplementary Materials & Methods Fig. 1 Isotype controls for Sema7a the APAP time course experiment

WT mice were fasted for 14 hours then treated with 350 mg/kg APAP, and culled at the indicated time points. As a control for the Sema7a DAB stain displayed in Fig. 1, a rabbit IgG isotype control stain was performed, for each time point, at the same time and concentration as the Sema7a antibody. Scale bars 100 µm.

Supplementary Materials & Methods Fig. 2 Controls for immunofluorescent stains

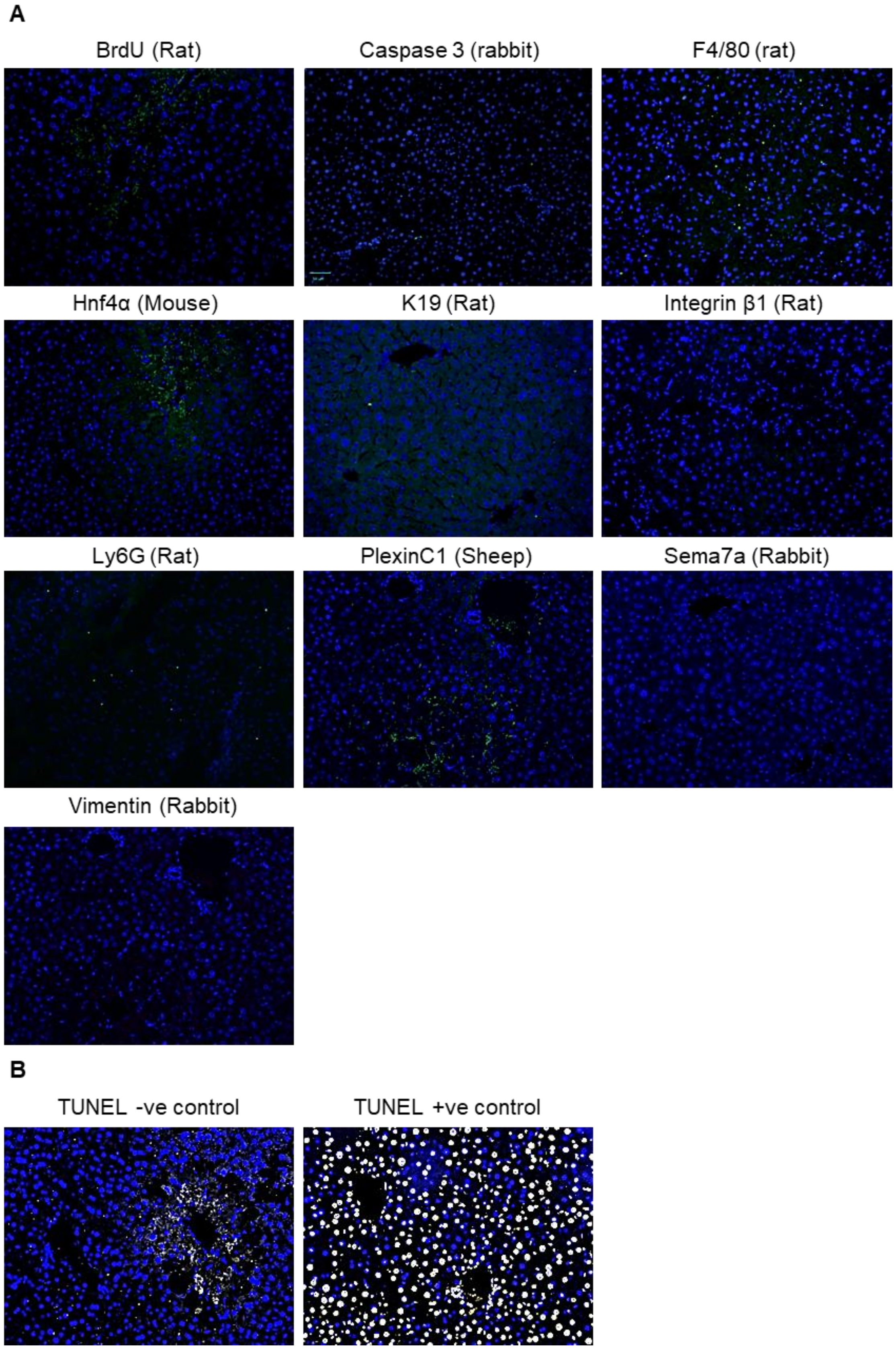

(A) Immunofluorescent isotype control images. Image labels indicate the corresponding immunostain for each isotype control. DNA is stained with DAPI. The fluorescent signal has been coloured green. 20x magnification

(B) Negative (left) and positive (right) controls for the TUNEL assay. The negative control used a 24 hour APAP injured tissue section, but without the enzyme required to label double stranded DNA breaks. Positive control used a healthy mouse, which was treated with DNAse I, as per manufacturer's instructions.

Supplementary Materials & Methods Fig. 3 Isotype controls for DAB stains

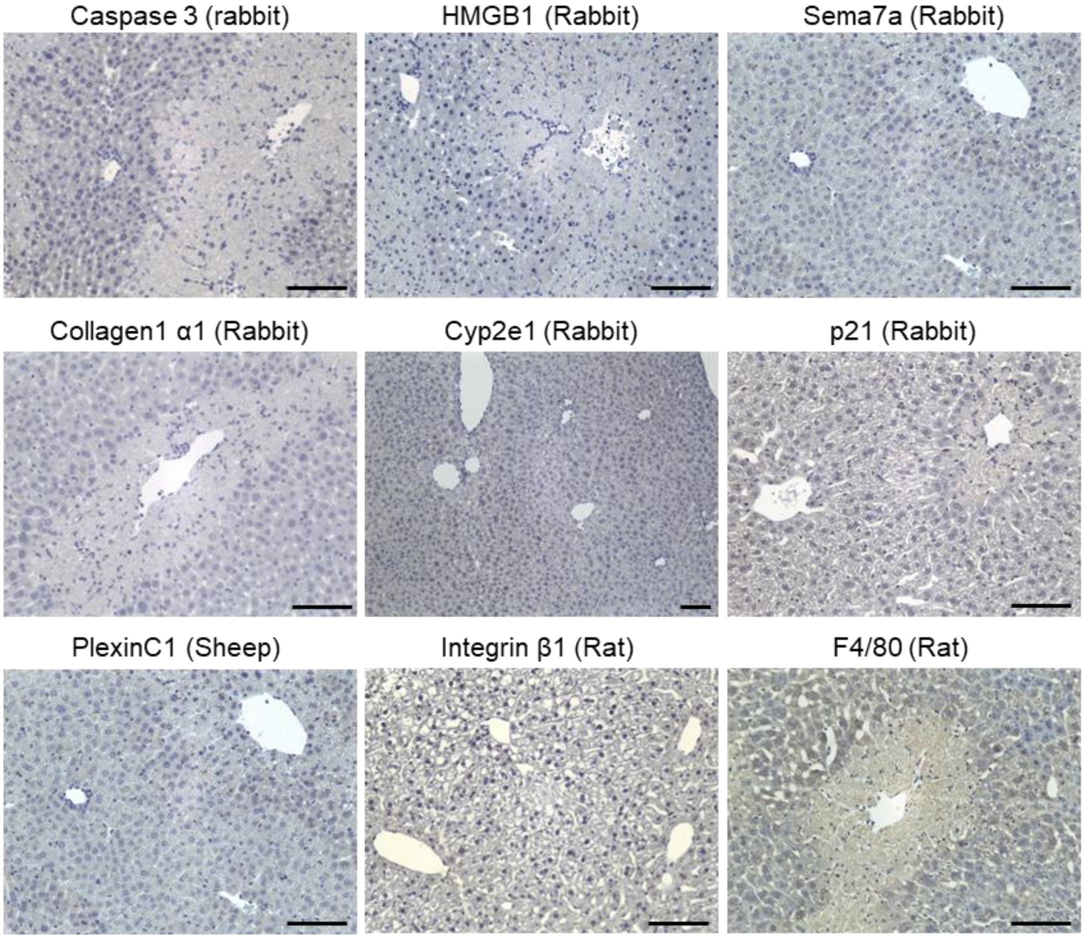

Brightfield images of isotype controls for DAB stains. Image labels indicate which immunostain the isotype control corresponds too. DNA is stained with haematoxylin. Scale bars 100 µm.

Image Analysis

Quantification of necrotic area

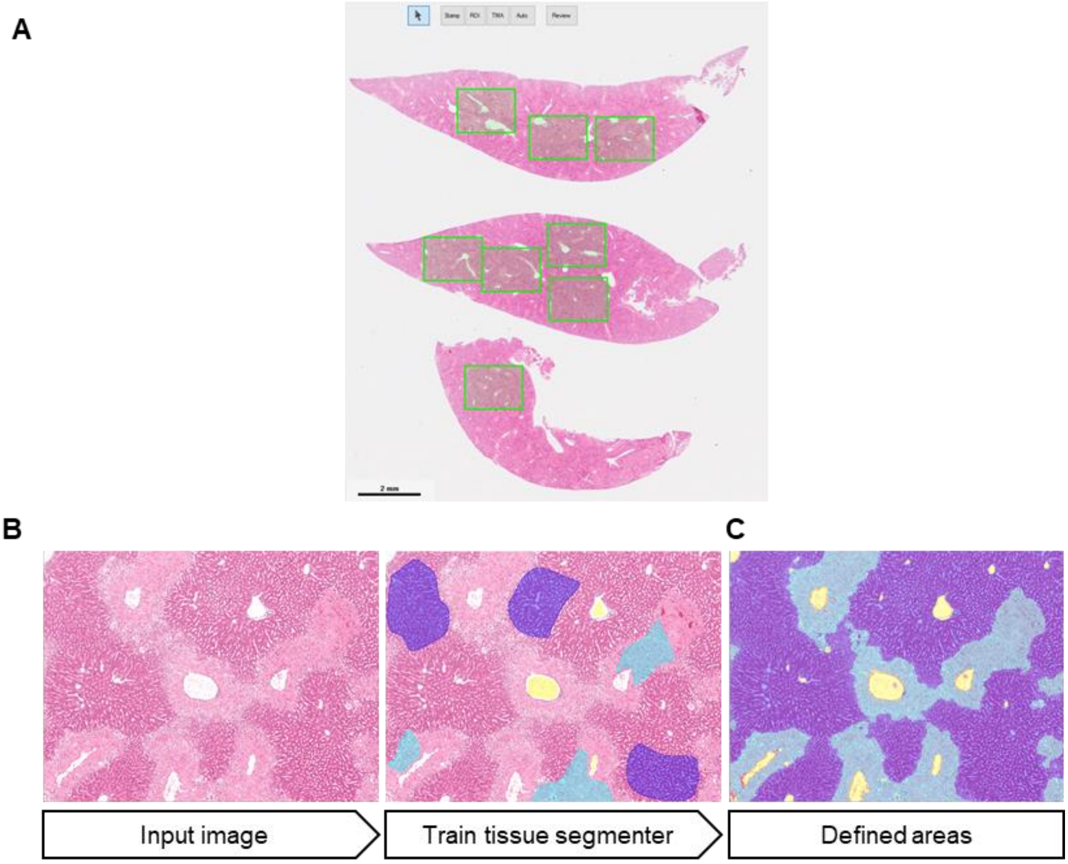

Supplementary Materials & Methods Fig. 4 Necrosis analysis using the inForm software

Example pipeline to analyse necrosis using the Polaris and the inForm software

(A) Eight 10x FOV were selected per HnE stained liver section. The Polaris uses MSI to image the selected fields. Scale bar 2 mm.

(B) To segment the image, the inForm software was trained to identify: necrosis (cyan), healthy parenchyma (purple), vessels (yellow) and red blood cells (RBCs, red).

(C) After training, the inForm software reliably segments the tissue into the defined areas, which can then be used to calculate %necrosis.

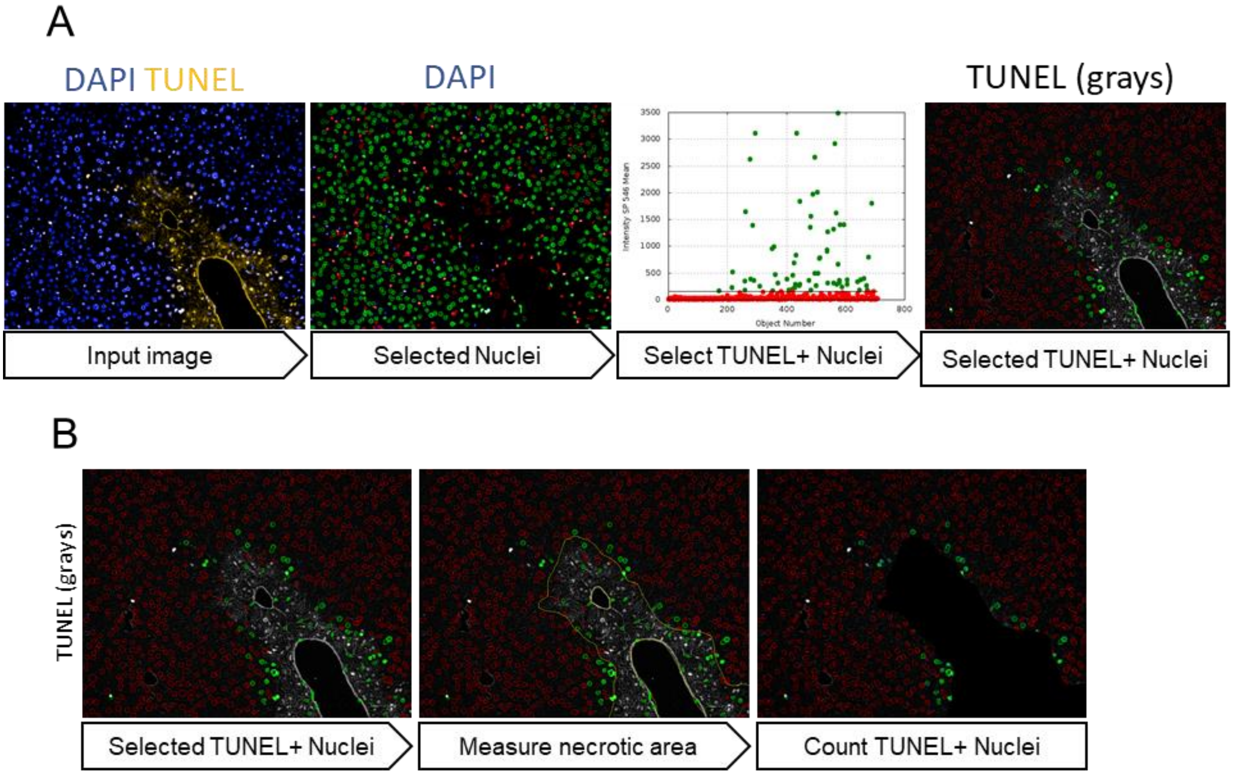

Supplementary Materials & Methods Fig. 5 Strategy to quantify TUNEL+ DAPI+ cells

The Perkin Elmer Operetta microscope and Columbus software were used to image and detect TUNEL positive nuclei. ImageJ software was used to count TUNEL+ cells, outside the necrotic zone

(A) Nuclei were selected using the following: intensity 452<2300; roundness >0.82; nuclear area >47. TUNEL+ nuclei were subsequently identified using fluorescence intensity <400 (second right).

(B) Images from Columbus were analysed in ImageJ (left). The necrotic area was measured (yellow, center) and removed (right). Remaining TUNEL+ nuclei (green) were counted using the Cell Counter plugin (right).

DAPI, blue; TUNEL, yellow; Green, selected nuclei; Red, unselected nuclei. x20 magnification

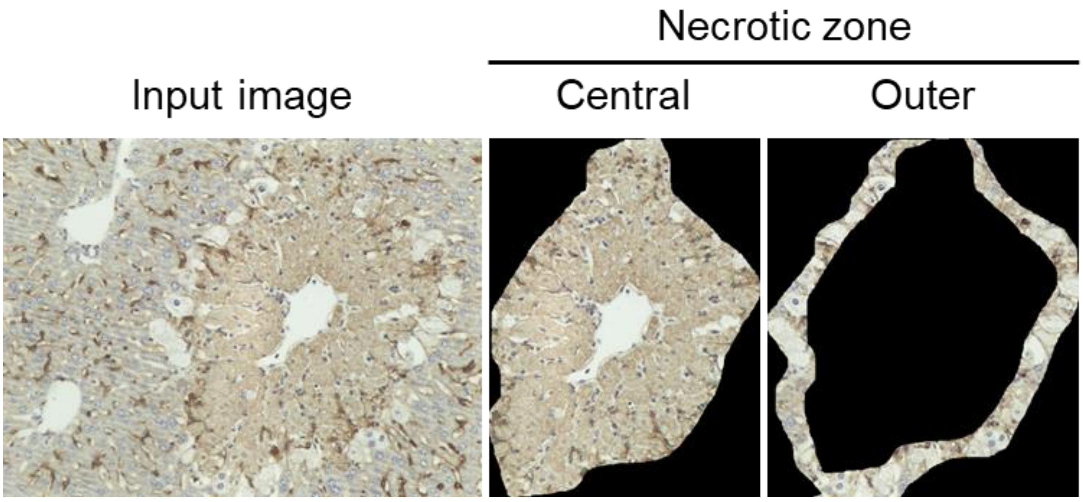

Supplementary Materials & Methods Fig. 6 Quantification of the number of F4/80+ cells per area

F4/80 DAB stained 20x images from both WT and Sema7a KO mice were analysed using Fiji ImageJ. First the central necrotic zone was selected and removed from the input image. Then the outer necrotic zone was selected and separated from the input image. F4/80+ macrophages were manually counted in the healthy parenchyma, central and outer necrotic zone and dived by the area (pixels ^{2}), to give the number of F4/80+ macrophages per area.

Flow cytometry

Flow cytometry on peripheral blood

Peripheral blood was collected into anticoagulating EDTA tubes from WT or Sema7a KO mice through a tail nick or the IVC. 25 µL blood was stained for 20 min at RT in the required antibody cocktail.

Cells were washed with PBS and stained with 1:1,000 Live/ Dead (L/D) e780 (L10120, Life technologies) for 40 min, followed by fixation and lysis with 200 µL BD Phosflow Lyse/ Fix (BD Biosciences) for 15 min RT. Cells were kept in FACS wash (PBS+ 2% FBS).

Cells were gated on size, singlets, live, CD45+, and Lineage- (B and T cell exclusion). Gates were set using FMOs.

Sema7a receptor expression on neutrophils

Peripheral blood was stained with the panel in Supplementary Table 3 and analysed on the Novocyte with NovoExpress 1.2.4 software (ACEA Biosciences). Cells were gated for size, singlets, L/D-, CD45+ and Ly6G+. This neutrophil population was examined for the expression of the Sema7a receptors: Integrinα1 and Integrinαυ, Plexin C1. All gates were set using FMOs.

Supplementary Table 3 Sema7a receptor panel on peripheral blood

Marker	Fluorophore	Clone	Channel	Manufacturer	Staining Conc.
CD11b	BV650	M1/70	V675/30	Biolegend	1:100
Integrin α1	APC	HMα1	R675/30	Biolegend	1:100
Integrin αυ	PE	RMV-7	B572/28	Biolegend	1:100
Plexin C1	Alexa 488	-	B530/30	R&D	1:50
Ly6G	Pacific Blue	1A8	V445/45	Biolegend	1:100
Lineage (CD3, CD19,)	PE Cy7	CD3: 145-2C11<br>CD19: 6D5	B780/60	Biolegend<br>Biolegend	1:200
L/D	e780		R780/60	Life technologies	1:1,000

Numbers of neutrophils and monocytes in peripheral blood

Peripheral blood was isolated from healthy 10-12 week old male WT and Sema7a KO mice, and stained with the panel in Supplementary Table 4. From the singlet, live, CD45+, Lineage- population Ly6G+ neutrophils and Ly6G- monocytes were selected. Monocytes were further subdivided in to Ly6C^{hi} and Ly6C ^{lo}. Data was analysed on the Novocyte, (ACEA Biosciences). Data was quantified using NovoExpress 1.2.4 software (ACEA Biosciences).:

Supplementary Table 4 Neutrophil and monocyte panel for peripheral blood

Marker	Fluorophore	Clone	Channel	Manufacturer	Staining Conc.
CD45	BV650	30-F11	V675/30	Biolegend	1:100
CD11b	Alexa Flour 488	M1/70	B530/30	eBioscience	1:100
CD115	APC	AFS98	R675/30	Biolegend	1:100
Ly6G	PE	1A8	B572/28	Biolegend	1:100
Ly6C	PB	HK1.4	V445/45	Biolegend	1:100
Lineage (CD3, CD19,)	PE Cy7	CD3: 145-2C11<br>CD19: 6D5	B780/60	Biolegend<br>Biolegend	1:200
L/D	e780		R780/60	Life technologies	1:1,000

In vivo phagocytosis flow cytometry analysis of blood

10-12 week old WT and Sema7a KO mice were starved for 12 hours and injected with 350 mg/kg APAP. 8 hours later, mice were administered PKH, a phagocytic dye. At 24 hours post APAP injection, mice were humanely culled and blood was collected from mice through a cardiac puncture. 30 µL blood was stained with the cocktail in Supplementary Table 5 or FMO equivalent, and analysed on the BD LSR Fortessa SORP FACS. Ly6G+ neutrophils and Ly6C^{hi} and Ly6C^{lo} monocytes were selected from the CD45+ Lineage- population and analysed for frequency of phagocytosis (%PKH+). All gates were set using an FMO.

Supplementary Table 5 In vivo phagocytosis flow cytometry panel for blood

Marker	Fluorophore	Clone	Channel	Manufacturer	Staining Conc.
CD45	BV650	30-F11	V660/20	Biolegend	1:100
CD11b	Alexa Flour 488	M1/70	B530/30	eBioscience	1:100
CD115	APC	AFS98	R670/14	Biolegend	1:100
Ly6G	PB	1A8	V450/50	Biolegend	1:100
Ly6C	PerCyP/Cy5.5	HK1.4	B710/50	Biolegend	1:100
Lineage (CD3, CD19,)	PE Cy7	CD3: 145-2C11<br>CD19: 6D5	Y/G 780/60	Biolegend<br>Biolegend	1:200
L/D	e780		R780/60	Life technologies	1:1,000
PKH	Fluoresces in the PE channel		Y/G 586/15	Sigma	

Liver digestion and non-parenchymal cell isolation and staining for flow cytometry

Isolation of hepatic non-parenchymal cells (NPCs), protocol followed Campana's [1] protocol, with minor modifications.

0.5 g of perfused liver from the central lobe was mechanically homogenised with a scalpel, followed by enzymatic digestion: collagenase V (0.8 mg/ml; Sigma-Aldrich), collagenase D (0.625 mg/ml; Roche), dispase (1 mg/ml, Gibco), and DNase (30 µg/ml, Roche) in RPMI 1640, for 20 min at 37 °C in a shaking incubator, with vigorous shaking every 5 min.

Liver digests were filtered through a 100 µm filter, and enzymes inactivated by adding 30 ml cold medium (RPMI,10% FCS, 4 °C). The NPC fraction of the liver was harvested by two centrifugations in 30 ml cold medium at 300 x g for 5 min at 4 °C, discarding the supernatant each time.

NPC suspensions were passed through a 40 µm filter, stained with toluidine blue dye and live cells were counted with a BioRad analyser, then diluted to 7,000,000 cells/ml.

NPCs were washed in PBS and stained with Live/Dead (L/D) for 40 min at RT, then blocked in 10% mouse serum. 1,000,000 cells/sample were stained with the Liver PKH Flow Cytometry Panel (Supplementary Table 6). for 40 min Room Temperature (RT) followed by two FACS buffer washes.

Red blood cells were lysed, and NPCs were fixed with 1x BD Phosflow Lyse/ Fix (BD Biosciences) for 10 min at 4 °C. The NPC suspension was washed and kept in FACS buffer.

Calculating the absolute number of NPCs per gram of liver

The total number of live cells in the whole liver digest volume was calculated and divided by the weight of the liver lobe before digestion, providing the number of live cells per gram of liver. Each specific NPC population count was made into a percentage of the live cell count,

as analysed by the BD LSR Fortessa SORP FACS. This was multiplied by the number of live cells per gram of liver, giving the absolute number of a specific NPC population per gram of liver.

NPC staining for flow cytometry and in vivo phagocytosis

WT and Sema7a KO mice were injected with APAP. 8 hours later mice were injected with PKH. Mice were humanely culled at 12 or 24 hours 350mg/kg APAP**.** The NPC fraction of the liver was stained with the antibodies in Supplementary Table 6. Neutrophils, monocytes and macrophages were identified as follows: the NPC fraction was selected through cells, singlets, live (L/D-), CD45+, and Lineage–. From this population Ly6G+ (neutrophils), Ly6G- CD11b^{lo}, F4/80^{hi} liver resident macrophages (Mϕ) and Ly6G, CD11b+, F4/80^{lo} infiltrating Mϕ were selected. The infiltrating Mϕ were further subdivided to Ly6C^{hi} and Ly6C^{lo} populations. Each NPC population was analysed for phagocytosis (PKH positivity). Gates were set using FMOs.

Supplementary Table 6 In vivo phagocytosis flow cytometry panel for NPCs of the liver

Marker	Fluorophore	Clone	Channel	Manufacturer	Staining Conc.
CD45	AF700	30-F11	R730/45	Biolegend	1:100
CD11b	BV650	M1/70	V670/30	eBioscience	1:100
Ly6G	PB	1A8	V450/50	BD Pharmingen	1:100
CD62L	FITC		B530/30		1:200
F4/80	APC	BM8	R670/30	Biolegend	1:100
Ly6C	PerCP/Cy5.5	HK1.4	B695/40	Biolegend	1:100
DUMP (CD3, CD19, NK1.1)	PE/Cy7	CD3=145-2C11, CD19=6D5	Y/G 780/60	Biolegend	1:200
L/D	e780		R780/60	Life technologies	1:1,000
PKH	Fluoresces in the PE channel		Y/G 582/15	Sigma	

References

1. Campana, L. *et al.* The STAT3–IL-10–IL-6 Pathway Is a Novel Regulator of Macrophage Efferocytosis and Phenotypic Conversion in Sterile Liver Injury. *J. Immunol.* **200**, 1169–1187 (2018).
